# Supplementary material for: Match analysis and probability of winning a point in elite men’s singles tennis
Source: PLoS One. 2023 Sep 28;18(9):e0286076. doi: 10.1371/journal.pone.0286076 (PMC10538650; doi:10.1371/journal.pone.0286076)
Supplement: S1 Table — (DOCX) [file pone.0286076.s001.docx]

**S1Table**

Description of variables that affect performance on the different court surfaces in the 2021 season, analysis of the distribution of categories of each variable by surface (intra-variable χ2) and comparative analysis between court surfaces (χ2 inter-variable).

|  |  | **Clay (RG)** | | **X2 Intra-variable RG** | **Grass (WI)** | | **X2 Intra-variable WI** | **Hard (US)** | | **X2 Intra-variable US** | **X2 Inter-variable** | **Coef.Contig.** |
| --- | --- | --- | --- | --- | --- | --- | --- | --- | --- | --- | --- | --- |
|  |  | n | % |  | n | % |  | n | % |  |  |  |
| **SERVICE** | DF | 45 | 2,7 | χ2=970,859 | 60 | 3,7 | χ2=822,858 | 58 | 4,2 | χ2=735,654 | χ2=8,174 | .042 |
|  | FS | 1081 | 65,1 | *p*=.000 | 1003 | 61,8 | *p*=.000 | 882 | 63,6 | *p*=.000 | *p*=.085 |  |
|  | SS | 534 | 32,2 |  | 506 | 34,5 |  | 446 | 32,2 |  |  |  |
| **RALLY LENGHT** | LN | 223 | 13,4 | χ2=762,941 | 112 | 6,9 | χ2=1440,048 | 182 | 13,1 | χ2=790,926 | χ2=74,200 | .125 |
|  | MD | 359 | 21,6 | *p*=.000 | 254 | 15,7 | *p*=.000 | 250 | 18,0 | *p*=.000 | *p*=.000 |  |
|  | SH | 1078 | 64,9 |  | 1257 | 77,4 |  | 954 | 68,8 |  |  |  |
| **BOUNCE ZONE** | SZ | 141 | 8,5 | χ2=1135,906 | 187 | 11,5 | χ2=1431,325 | 213 | 15,4 | χ2=910,745 | χ2=66,987 | .119 |
|  | ZB1 | 711 | 42,8 | *p*=.000 | 797 | 49,1 | *p*=.000 | 596 | 43,0 | *p*=.000 | *p*=.000 |  |
|  | ZB2 | 441 | 26,6 |  | 339 | 20,9 |  | 313 | 22,6 |  |  |  |
|  | ZB3 | 197 | 11,9 |  | 186 | 11,5 |  | 164 | 11,8 |  |  |  |
|  | ZB4 | 99 | 6,0 |  | 56 | 3,5 |  | 57 | 4,1 |  |  |  |
|  | ZB5 | 71 | 4,3 |  | 58 | 3,6 |  | 43 | 3,1 |  |  |  |
| **THE FINISH ZONE** | BSO | 278 | 16,7 | χ2=429,080 | 234 | 14,4 | χ2=899,829 | 218 | 15,7 | χ2=775,455 | χ2=87,508 | .136 |
|  | LTO | 229 | 13,8 | *p*=.000 | 155 | 9,6 | *p*=.000 | 121 | 8,7 | *p*=.000 | *p*=.000 |  |
|  | NET | 279 | 16,8 |  | 332 | 20,5 |  | 269 | 19,4 |  |  |  |
|  | Z1 | 400 | 24,1 |  | 534 | 32,9 |  | 460 | 33,2 |  |  |  |
|  | Z2 | 94 | 5,7 |  | 103 | 6,3 |  | 81 | 5,8 |  |  |  |
|  | Z3 | 56 | 3,4 |  | 42 | 2,6 |  | 34 | 2,5 |  |  |  |
|  | Z4 | 135 | 8,1 |  | 97 | 6,0 |  | 105 | 7,6 |  |  |  |
|  | Z5 | 189 | 11,4 |  | 126 | 7,8 |  | 98 | 7,1 |  |  |  |
| **WINNER** | RW | 615 | 37,0 | χ2=111,386 | 565 | 34,8 | χ2=149,753 | 489 | 35,3 | χ2=120,104 | χ2=1,972 | .021 |
|  | SW | 1045 | 63,0 | *p*=.000 | 1058 | 65,2 | *p*=.000 | 897 | 64,7 | *p*=.000 | *p*=.373 |  |
| **POINT ENDING** | RWFE | 89 | 5,4 | χ2=363,692 | 62 | 3,8 | χ2=407,547 | 58 | 4,2 | χ2=328,242 | χ2=26,960 | .076 |
|  | RWUE | 393 | 23,7 | *p*=.000 | 402 | 24,8 | *p*=.000 | 334 | 24,1 | *p*=.000 | *p*=.003 |  |
|  | RWW | 133 | 8,0 |  | 101 | 6,2 |  | 97 | 7,0 |  |  |  |
|  | SWFE | 246 | 14,8 |  | 335 | 20,6 |  | 252 | 18,2 |  |  |  |
|  | SWUE | 392 | 23,6 |  | 358 | 22,1 |  | 314 | 22,7 |  |  |  |
|  | SWW | 407 | 24,5 |  | 365 | 22,5 |  | 331 | 23,9 |  |  |  |
| **FINISH AND FINAL STROKE** | RDF | 38 | 2,3 | χ2=866,120 | 60 | 3,7 | χ2=858,121 | 58 | 4,2 | χ2=830,335 | χ2=172,046 | .189 |
|  | RFEBH | 23 | 1,4 | *p*=.000 | 22 | 1,4 | *p*=.000 | 25 | 1,8 | *p*=.000 | *p*=.000 |  |
|  | RFEFH | 61 | 3,7 |  | 30 | 1,8 |  | 29 | 2,1 |  |  |  |
|  | RFEOT | 5 | ,3 |  | 10 | 0,6 |  | 5 | 0,4 |  |  |  |
|  | RUEBH | 133 | 8,0 |  | 77 | 4,7 |  | 105 | 7,6 |  |  |  |
|  | RUEFH | 183 | 11,0 |  | 196 | 12,1 |  | 150 | 10,8 |  |  |  |
|  | RUEOT | 40 | 2,4 |  | 69 | 4,3 |  | 20 | 1,4 |  |  |  |
|  | RWBH | 28 | 1,7 |  | 24 | 1,5 |  | 26 | 1,9 |  |  |  |
|  | RWFH | 73 | 4,4 |  | 42 | 2,6 |  | 50 | 3,6 |  |  |  |
|  | RWOT | 31 | 1,9 |  | 35 | 2,2 |  | 21 | 1,5 |  |  |  |
|  | SACE | 99 | 6,0 |  | 125 | 7,7 |  | 155 | 11,2 |  |  |  |
|  | SFEBH | 92 | 5,5 |  | 152 | 9,4 |  | 101 | 7,3 |  |  |  |
|  | SFEFH | 139 | 8,4 |  | 166 | 10,2 |  | 137 | 9,9 |  |  |  |
|  | SFEOT | 16 | 1,0 |  | 17 | 1,0 |  | 14 | 1,0 |  |  |  |
|  | SUEBH | 172 | 10,4 |  | 135 | 8,3 |  | 145 | 10,5 |  |  |  |
|  | SUEFH | 193 | 11,6 |  | 181 | 11,2 |  | 157 | 11,3 |  |  |  |
|  | SUEOT | 26 | 1,6 |  | 42 | 2,6 |  | 12 | 0,9 |  |  |  |
|  | SWBH | 51 | 3,1 |  | 22 | 1,4 |  | 28 | 2,0 |  |  |  |
|  | SWFH | 168 | 10,1 |  | 112 | 6,9 |  | 89 | 6,4 |  |  |  |
|  | SWOT | 89 | 5,4 |  | 106 | 6,5 |  | 59 | 4,3 |  |  |  |

*Note. Abbreviations in table 1. RG: Roland Garros; Wi: Wimbledon; US: US Open*
