# Supplementary material for: Match analysis and probability of winning a point in elite men’s singles tennis
Source: PLoS One. 2023 Sep 28;18(9):e0286076. doi: 10.1371/journal.pone.0286076 (PMC10538650; doi:10.1371/journal.pone.0286076)
Supplement: S2 Table — (DOCX) [file pone.0286076.s002.docx]

**S2 Table**

Analysis of different combinations of variables that affect performance (service, rally length, bounce zone, the finish zone and point ending) as a function of the court surface.

| **Clay (RG)** | | | **SWW** | | | | | | | **SWFE** | | | | | | | **SWUE** | | | | | **RWW** | | | | | | | **RWFE** | | | | | | | **RWUE** | | | | |
| --- | --- | --- | --- | --- | --- | --- | --- | --- | --- | --- | --- | --- | --- | --- | --- | --- | --- | --- | --- | --- | --- | --- | --- | --- | --- | --- | --- | --- | --- | --- | --- | --- | --- | --- | --- | --- | --- | --- | --- | --- |
| **N=1520** |  |  |  |  | **Z1** | **Z2** | **Z3** | **Z4** | **Z5** |  |  | **Z1** | **Z2** | **Z3** | **Z4** | **Z5** |  |  | **BS** | **LT** | **NT** |  |  | **Z1** | **Z2** | **Z3** | **Z4** | **Z5** |  |  | **Z1** | **Z2** | **Z3** | **Z4** | **Z5** |  |  | **BS** | **LT** | **NT** |
| **Patterns** | **N** | **%** | **N** | **%** | **%** | **%** | **%** | **%** | **%** | **N** | **%** | **%** | **%** | **%** | **%** | **%** | **N** | **%** | **%** | **%** | **%** | **N** | **%** | **%** | **%** | **%** | **%** | **%** | **N** | **%** | **%** | **%** | **%** | **%** | **%** | **N** | **%** | **%** | **%** | **%** |
| FS-SH-ZB1 | 373 | 25 | 85 | 23 | 41 | 9 | 9 | 12 | 28 | 112 | 30 | 88 | 3 | 1 | 4 | 5 | 123 | 33 | 52 | 18 | 30 | 12 | 3 | 33 | 17 |  |  | 50 | 13 | 3 | 15 | 15 | 23 | 15 | 31 | 28 | 8 | 18 | 43 | 39 |
| FS-SH-ZB2 | 159 | 10 | 55 | 35 | 20 | 18 | 7 | 27 | 27 | 22 | 14 | 18 | 14 | 9 | 14 | 45 | 17 | 11 | 41 | 18 | 41 | 9 | 6 | 22 |  | 11 | 33 | 33 | 4 | 3 |  | 25 |  | 25 | 50 | 52 | 33 | 18 | 37 | 45 |
| FS-SH-ZB3 | 63 | 4 | 14 | 22 | 43 | 14 | 7 | 21 | 14 | 3 | 5 |  | 33 |  | 67 |  | 10 | 16 | 20 | 30 | 50 | 1 | 2 |  |  |  | 100 |  | 1 | 2 |  |  |  |  | 100 | 34 | 54 | 56 | 29 | 15 |
| FS-SH-ZB4 | 26 | 2 | 5 | 19 | 40 |  |  | 20 | 40 | 4 | 15 | 25 |  |  | 25 | 50 | 5 | 19 | 60 |  | 40 | 1 | 4 |  |  |  |  | 100 | 1 | 4 |  |  | 100 |  |  | 10 | 38 | 20 | 20 | 60 |
| FS-SH-ZB5 | 17 | 1 | 2 | 12 |  |  |  | 100 |  | 1 | 6 | 100 |  |  |  |  | 7 | 41 | 43 | 57 |  | 2 | 12 | 50 |  |  | 50 |  | 1 | 6 |  |  |  | 100 |  | 4 | 24 | 25 | 50 | 25 |
| FS-MD-ZB1 | 79 | 5 | 16 | 20 | 6 | 2 | 1 | 2 | 5 | 15 | 19 | 20 | 20 | 20 | 13 | 27 | 14 | 18 | 14 | 29 | 57 | 20 | 25 | 45 | 15 |  | 25 | 15 | 7 | 9 | 43 |  |  | 29 | 29 | 7 | 9 | 43 |  | 57 |
| FS-MD-ZB2 | 73 | 5 | 13 | 18 | 23 | 23 |  | 31 | 23 | 5 | 7 |  | 20 | 20 | 40 | 20 | 19 | 26 | 37 | 21 | 42 | 9 | 12 | 11 |  | 22 | 33 | 33 | 8 | 11 | 13 | 25 | 13 | 25 | 25 | 19 | 26 | 42 | 16 | 42 |
| FS-MD-ZB3 | 29 | 2 | 3 | 10 | 67 |  |  | 33 |  | 4 | 14 | 25 |  |  | 25 | 50 | 4 | 14 | 25 | 25 | 50 | 2 | 7 | 100 |  |  |  |  | 2 | 7 |  |  | 50 | 50 |  | 14 | 48 | 50 | 43 | 7 |
| FS-MD-ZB4 | 23 | 2 | 1 | 4 | 100 |  |  |  |  | 1 | 4 |  |  |  |  | 100 | 5 | 22 | 60 | 20 | 20 | 4 | 17 | 25 |  |  |  | 75 | 2 | 9 |  |  | 50 |  | 50 | 10 | 43 | 50 |  | 50 |
| FS-MD-ZB5 | 11 | 1 | 1 | 9 | 100 |  |  |  |  | 0 | 0 |  |  |  |  |  | 5 | 45 | 60 | 20 | 20 | 2 | 18 |  |  |  | 100 |  | 0 | 0 |  |  |  |  |  | 3 | 27 |  | 67 | 33 |
| FS-LN-ZB1 | 36 | 2 | 12 | 33 | 58 | 8 | 8 | 17 | 8 | 4 | 11 | 50 |  |  | 25 | 25 | 5 | 14 |  | 60 | 40 | 7 | 19 | 57 |  |  | 14 | 29 | 5 | 14 | 40 | 20 |  | 20 | 20 | 3 | 8 | 33 |  | 67 |
| FS-LN-ZB2 | 46 | 3 | 10 | 22 | 30 |  | 10 | 30 | 30 | 8 | 17 | 50 |  |  | 25 | 25 | 11 | 24 | 27 | 55 | 18 | 6 | 13 | 50 | 33 |  | 17 |  | 3 | 7 | 33 | 33 |  |  | 33 | 8 | 17 | 38 | 25 | 38 |
| FS-LN-ZB3 | 28 | 2 | 5 | 18 | 20 |  | 20 | 20 | 40 | 5 | 18 | 40 | 20 |  | 20 | 20 | 5 | 18 | 40 | 60 |  | 2 | 7 | 50 | 50 |  |  |  | 1 | 4 | 100 |  |  |  |  | 10 | 36 | 40 | 20 | 40 |
| FS-LN-ZB4 | 12 | 1 | 2 | 17 | 50 |  |  |  | 50 | 2 | 17 |  | 50 |  | 50 |  | 6 | 50 | 67 | 17 | 17 | 1 | 8 |  |  |  |  | 100 |  | 0 |  |  |  |  |  | 1 | 8 |  | 100 |  |
| FS-LN-ZB5 | 12 | 1 | 2 | 17 |  |  |  |  | 100 | 3 | 25 | 67 |  |  |  | 33 | 3 | 25 | 33 | 33 | 33 | 3 | 25 | 67 |  | 33 |  |  | 1 | 8 |  |  |  | 100 |  |  | 0 |  |  |  |
| SS-SH-ZB1 | 145 | 10 | 12 | 8 | 25 |  | 8 | 25 | 42 | 11 | 8 | 73 | 18 |  |  | 9 | 70 | 48 | 50 | 26 | 24 | 15 | 10 | 27 | 7 | 7 | 7 | 53 | 16 | 11 | 25 |  | 19 | 38 | 19 | 21 | 14 | 33 | 33 | 33 |
| SS-SH-ZB2 | 83 | 5 | 17 | 20 | 24 | 12 |  | 35 | 29 | 8 | 10 | 25 | 13 |  | 25 | 38 | 15 | 18 | 13 | 13 | 73 | 3 | 4 | 33 |  |  |  | 67 | 3 | 4 |  | 33 |  |  | 67 | 37 | 45 | 22 | 35 | 43 |
| SS-SH-ZB3 | 45 | 3 | 4 | 9 | 75 |  |  |  | 25 | 3 | 7 | 67 |  | 33 |  |  | 8 | 18 | 25 | 50 | 25 | 2 | 4 |  |  |  | 50 | 50 |  | 0 |  |  |  |  |  | 28 | 62 | 36 | 39 | 25 |
| SS-SH-ZB4 | 13 | 1 | 3 | 23 | 33 |  |  |  | 67 | 1 | 8 | 100 |  |  |  |  | 2 | 15 |  | 50 | 50 |  | 0 |  |  |  |  |  | 1 | 8 |  | 100 |  |  |  | 6 | 46 | 50 | 33 | 17 |
| SS-SH-ZB5 | 14 | 1 | 3 | 21 |  | 33 | 33 | 33 |  | 2 | 14 |  |  |  |  | 100 | 3 | 21 |  | 67 | 33 | 1 | 7 |  |  |  | 100 |  | 2 | 14 |  |  |  |  | 100 | 3 | 21 | 33 | 33 | 33 |
| SS-MD-ZB1 | 47 | 3 | 16 | 34 | 56 | 19 |  | 19 | 6 | 4 | 9 | 25 | 25 | 50 |  |  | 9 | 19 | 44 | 33 | 22 | 7 | 15 | 43 | 29 |  | 14 | 14 | 1 | 2 |  |  |  | 100 |  | 10 | 21 | 20 | 40 | 40 |
| SS-MD-ZB2 | 49 | 3 | 9 | 18 |  | 11 | 22 | 11 | 56 | 10 | 20 | 30 | 20 |  | 20 | 30 | 14 | 29 | 29 | 29 | 43 | 5 | 10 | 20 | 20 |  | 60 |  | 4 | 8 |  |  | 50 |  | 50 | 7 | 14 | 14 | 57 | 29 |
| SS-MD-ZB3 | 21 | 1 | 1 | 5 |  |  |  | 100 |  | 5 | 24 | 20 | 20 | 20 | 20 | 20 | 6 | 29 | 33 | 17 | 50 | 2 | 10 |  |  |  | 50 | 50 | 3 | 14 |  |  |  | 67 | 33 | 4 | 19 | 25 | 75 |  |
| SS-MD-ZB4 | 14 | 1 |  | 0 |  |  |  |  |  |  | 0 |  |  |  |  |  | 4 | 29 | 50 | 25 | 25 | 3 | 21 | 33 |  |  |  | 67 |  | 0 |  |  |  |  |  | 7 | 50 | 14 | 57 | 29 |
| SS-MD-ZB5 | 13 | 1 | 1 | 8 | 100 |  |  |  |  |  | 0 |  |  |  |  |  | 3 | 23 | 33 | 33 | 33 | 1 | 8 |  |  |  | 100 |  | 3 | 23 | 33 |  |  | 33 | 33 | 5 | 38 | 100 |  |  |
| SS-LN-ZB1 | 31 | 2 | 10 | 32 | 60 | 10 | 20 | 10 |  | 3 | 10 | 67 | 33 |  |  |  | 3 | 10 |  | 67 | 33 | 9 | 29 | 44 | 11 |  | 11 | 33 | 2 | 6 |  |  | 50 | 50 |  | 4 | 13 |  | 25 | 75 |
| SS-LN-ZB2 | 32 | 2 | 5 | 16 | 20 |  | 20 | 40 | 20 | 8 | 25 | 38 |  | 38 | 13 | 13 | 9 | 28 | 33 | 22 | 44 | 1 | 3 |  |  |  |  | 100 | 2 | 6 | 50 |  |  |  | 50 | 7 | 22 | 29 | 29 | 43 |
| SS-LN-ZB3 | 11 | 1 | 3 | 27 | 67 |  |  |  | 33 |  | 0 |  |  |  |  |  | 5 | 45 | 20 | 60 | 20 | 1 | 9 |  |  |  |  | 100 | 1 | 9 |  |  |  |  | 100 | 1 | 9 |  |  | 100 |
| SS-LN-ZB4 | 11 | 1 | 2 | 18 | 50 |  |  |  | 50 |  | 0 |  |  |  |  |  | 2 | 18 | 50 |  | 50 |  | 0 |  |  |  |  |  | 2 | 18 |  |  | 100 |  |  | 5 | 45 |  | 80 | 20 |
| SS-LN-ZB5 | 4 | 0 |  | 0 |  |  |  |  |  | 1 | 25 |  | 100 |  |  |  |  | 0 |  |  |  | 2 | 50 | 50 |  |  | 50 |  |  | 0 |  |  |  |  |  | 1 | 25 | 100 |  |  |

S2 Table. Continued.

| **Grass (Wimbledon)** | | | **SWW** | | | | | | | **SWFE** | | | | | | | **SWUE** | | | | | **RWW** | | | | | | | **RWFE** | | | | | | | **RWUE** | | | | |
| --- | --- | --- | --- | --- | --- | --- | --- | --- | --- | --- | --- | --- | --- | --- | --- | --- | --- | --- | --- | --- | --- | --- | --- | --- | --- | --- | --- | --- | --- | --- | --- | --- | --- | --- | --- | --- | --- | --- | --- | --- |
| **N=1436** |  |  |  |  | **Z1** | **Z2** | **Z3** | **Z4** | **Z5** |  |  | **Z1** | **Z2** | **Z3** | **Z4** | **Z5** |  |  | **BS** | **LT** | **NT** |  |  | **Z1** | **Z2** | **Z3** | **Z4** | **Z5** |  |  | **Z1** | **Z2** | **Z3** | **Z4** | **Z5** |  |  | **BS** | **LT** | **NT** |
| **Patterns** | **N** | **%** | **N** | **%** | **%** | **%** | **%** | **%** | **%** | **N** | **%** | **%** | **%** | **%** | **%** | **%** | **N** | **%** | **%** | **%** | **%** | **N** | **%** | **%** | **%** | **%** | **%** | **%** | **N** | **%** | **%** | **%** | **%** | **%** | **%** | **N** | **%** | **%** | **%** | **%** |
| FS-SH-ZB1 | 500 | 35 | 107 | 21 | 52 | 15 | 10 | 7 | 16 | 241 | 48 | 89 | 4 | 2 | 2 | 3 | 85 | 17 | 31 | 11 | 59 | 14 | 3 | 50 | 14 | 7 | 7 | 21 | 9 | 2 | 22 |  | 33 | 33 | 11 | 44 | 9 | 21 | 21 | 59 |
| FS-SH-ZB2 | 128 | 9 | 34 | 27 | 18 | 15 | 12 | 27 | 29 | 19 | 15 | 5 | 26 | 26 | 21 | 21 | 13 | 10 | 31 | 23 | 46 | 11 | 9 | 27 | 9 |  | 18 | 46 | 3 | 2 | 100 |  |  |  |  | 48 | 38 | 44 | 21 | 35 |
| FS-SH-ZB3 | 54 | 4 | 12 | 22 | 17 | 17 | 8 | 42 | 17 | 4 | 7 |  | 25 |  | 75 |  | 7 | 13 | 43 |  | 57 | 3 | 6 | 100 |  |  |  |  | 1 | 2 |  |  |  |  | 100 | 27 | 50 | 19 | 30 | 52 |
| FS-SH-ZB4 | 20 | 1 | 1 | 5 |  |  |  | 100 |  | 2 | 10 |  |  |  | 50 | 50 | 10 | 50 | 20 | 40 | 40 | 2 | 10 |  |  |  |  | 100 | 1 | 5 | 100 |  |  |  |  | 4 | 20 | 25 |  | 75 |
| FS-SH-ZB5 | 14 | 1 | 4 | 29 |  |  | 25 | 75 |  |  | 0 |  |  |  |  |  | 4 | 29 | 25 |  | 75 | 1 | 7 |  |  |  | 100 |  | 2 | 14 | 50 |  |  | 50 |  | 3 | 21 | 67 |  | 33 |
| FS-MD-ZB1 | 45 | 3 | 11 | 24 | 64 | 18 |  | 9 | 9 | 7 | 16 | 71 | 14 | 14 |  |  | 7 | 16 |  | 57 | 43 | 9 | 20 | 78 | 11 |  |  | 11 | 2 | 4 | 100 |  |  |  |  | 9 | 20 | 22 | 11 | 67 |
| FS-MD-ZB2 | 28 | 2 | 4 | 14 | 25 | 25 | 25 | 25 |  |  | 0 |  |  |  |  |  | 9 | 32 | 44 | 11 | 44 | 4 | 14 | 75 |  |  |  | 25 | 1 | 4 |  |  | 100 |  |  | 10 | 36 | 30 | 20 | 50 |
| FS-MD-ZB3 | 23 | 2 | 2 | 9 | 100 |  |  |  |  | 2 | 9 | 50 |  |  | 50 |  | 11 | 48 | 36 | 36 | 27 | 3 | 13 |  | 33 |  | 33 | 33 | 2 | 9 | 50 |  |  |  | 50 | 3 | 13 |  | 33 | 67 |
| FS-MD-ZB4 | 5 | 0 |  | 0 |  |  |  |  |  |  | 0 |  |  |  |  |  | 2 | 40 | 50 | 50 |  | 1 | 20 |  |  |  |  | 100 |  | 0 |  |  |  |  |  | 2 | 40 |  | 50 | 50 |
| FS-MD-ZB5 | 7 | 0 |  | 0 |  |  |  |  |  | 2 | 29 |  |  |  | 50 | 50 | 2 | 29 | 100 |  |  | 1 | 14 |  |  |  | 100 |  |  | 0 |  |  |  |  |  | 2 | 29 |  |  | 100 |
| FS-LN-ZB1 | 20 | 1 | 4 | 20 | 50 | 25 |  |  | 25 |  | 0 |  |  |  |  |  | 3 | 15 | 67 |  | 33 | 1 | 5 |  |  |  |  | 100 | 4 | 20 | 25 | 25 |  |  | 50 | 8 | 40 | 13 | 13 | 75 |
| FS-LN-ZB2 | 14 | 1 | 2 | 14 |  |  |  | 50 | 50 | 2 | 14 |  |  |  | 50 | 50 | 5 | 36 | 20 | 40 | 40 | 1 | 7 | 100 |  |  |  |  | 1 | 7 |  |  |  |  | 100 | 3 | 21 |  | 67 | 33 |
| FS-LN-ZB3 | 12 | 1 | 1 | 8 | 100 |  |  |  |  | 1 | 8 |  |  |  |  | 100 | 4 | 33 | 75 |  | 25 |  | 0 |  |  |  |  |  | 1 | 8 | 100 |  |  |  |  | 5 | 42 | 20 | 60 | 20 |
| FS-LN-ZB4 | 2 | 0 |  | 0 |  |  |  |  |  |  | 0 |  |  |  |  |  |  | 0 |  |  |  |  | 0 |  |  |  |  |  | 1 | 50 |  |  |  |  | 100 | 1 | 50 | 100 |  |  |
| FS-LN-ZB5 | 5 | 0 |  | 0 |  |  |  |  |  | 1 | 20 |  |  |  | 100 |  | 3 | 60 | 33 |  | 67 |  | 0 |  |  |  |  |  |  | 0 |  |  |  |  |  | 1 | 20 | 100 |  |  |
| SS-SH-ZB1 | 165 | 11 | 14 | 8 | 7 | 36 | 7 | 21 | 29 | 23 | 14 | 78 | 4 | 4 | 4 | 9 | 76 | 46 | 43 | 25 | 32 | 14 | 8 | 21 | 14 | 7 | 7 | 50 | 11 | 7 | 27 | 18 |  | 36 | 18 | 27 | 16 | 33 | 15 | 52 |
| SS-SH-ZB2 | 102 | 7 | 12 | 12 | 17 | 25 |  | 17 | 42 | 7 | 7 | 14 | 14 | 14 | 43 | 14 | 22 | 22 | 50 | 23 | 27 | 4 | 4 | 25 | 25 |  | 50 |  | 5 | 5 | 20 |  |  | 20 | 60 | 52 | 51 | 25 | 31 | 44 |
| SS-SH-ZB3 | 56 | 4 | 3 | 5 | 33 |  |  | 33 | 33 |  | 0 |  |  |  |  |  | 21 | 38 | 62 | 14 | 24 |  | 0 |  |  |  |  |  | 1 | 2 |  |  |  |  | 100 | 31 | 55 | 32 | 26 | 42 |
| SS-SH-ZB4 | 17 | 1 | 2 | 12 | 100 |  |  |  |  | 1 | 6 | 100 |  |  |  |  | 4 | 24 |  | 25 | 75 | 1 | 6 |  |  |  |  | 100 | 3 | 18 | 33 |  | 33 |  | 33 | 6 | 35 | 33 | 33 | 33 |
| SS-SH-ZB5 | 14 | 1 | 2 | 14 | 50 |  |  |  | 50 | 1 | 7 |  |  |  | 100 |  | 5 | 36 | 60 | 20 | 20 |  | 0 |  |  |  |  |  | 1 | 7 | 100 |  |  |  |  | 5 | 36 | 40 |  | 60 |
| SS-MD-ZB1 | 48 | 3 | 9 | 19 | 56 | 11 |  | 11 | 22 | 5 | 10 | 60 | 20 |  | 20 |  | 6 | 13 | 33 | 17 | 50 | 17 | 35 | 47 | 18 |  | 24 | 12 | 2 | 4 | 50 | 50 |  |  |  | 9 | 19 | 44 | 22 | 33 |
| SS-MD-ZB2 | 47 | 3 | 3 | 6 |  |  | 67 |  | 33 | 4 | 9 |  |  |  | 25 | 75 | 21 | 45 | 43 | 19 | 38 | 6 | 13 | 17 |  |  | 50 | 33 | 2 | 4 | 50 |  |  |  | 50 | 11 | 23 | 18 | 27 | 55 |
| SS-MD-ZB3 | 29 | 2 | 1 | 3 |  |  |  |  | 100 | 2 | 7 | 50 | 50 |  |  |  | 11 | 38 | 36 | 18 | 46 |  | 0 |  |  |  |  |  | 3 | 10 | 67 | 33 |  |  |  | 12 | 41 | 50 | 33 | 17 |
| SS-MD-ZB4 | 10 | 1 |  | 0 |  |  |  |  |  | 2 | 20 | 100 |  |  |  |  | 4 | 40 | 50 |  | 50 | 1 | 10 |  |  |  |  | 100 | 1 | 10 |  | 100 |  |  |  | 2 | 20 |  |  | 100 |
| SS-MD-ZB5 | 12 | 1 | 3 | 25 | 33 |  |  | 67 |  | 1 | 8 | 100 |  |  |  |  | 2 | 17 | 50 |  | 50 | 2 | 17 |  | 50 |  |  | 50 | 2 | 17 |  |  |  |  | 100 | 2 | 17 |  | 100 |  |
| SS-LN-ZB1 | 19 | 1 | 4 | 21 | 50 | 50 |  |  |  | 2 | 11 | 50 |  |  | 50 |  | 4 | 21 | 25 | 25 | 50 | 2 | 11 | 50 |  |  | 50 |  | 3 | 16 | 33 | 33 |  | 33 |  | 4 | 21 |  | 50 | 50 |
| SS-LN-ZB2 | 20 | 1 | 2 | 10 |  |  | 50 |  | 50 | 2 | 10 |  |  |  |  | 100 | 9 | 45 | 22 | 33 | 44 | 1 | 5 |  |  |  | 100 |  |  | 0 |  |  |  |  |  | 6 | 30 |  | 33 | 67 |
| SS-LN-ZB3 | 12 | 1 | 1 | 8 |  |  |  |  | 100 | 2 | 17 |  |  |  | 50 | 50 | 5 | 42 | 60 |  | 40 | 1 | 8 |  |  |  |  | 100 |  | 0 |  |  |  |  |  | 3 | 25 |  | 33 | 67 |
| SS-LN-ZB4 | 2 | 0 |  | 0 |  |  |  |  |  | 1 | 50 |  |  |  |  | 100 |  | 0 |  |  |  |  | 0 |  |  |  |  |  |  | 0 |  |  |  |  |  | 1 | 50 |  |  | 100 |
| SS-LN-ZB5 | 6 | 0 | 1 | 17 |  |  |  | 100 |  |  | 0 |  |  |  |  |  | 3 | 50 | 100 |  |  | 1 | 17 |  |  |  | 100 |  |  | 0 |  |  |  |  |  | 1 | 17 |  |  | 100 |

Continúa

S2 Table. Continued.

| **Hard court (US)** | | | **SWW** | | | | | | | **SWFE** | | | | | | | **SWUE** | | | | | **RWW** | | | | | | | **RWFE** | | | | | | | **RWUE** | | | | |
| --- | --- | --- | --- | --- | --- | --- | --- | --- | --- | --- | --- | --- | --- | --- | --- | --- | --- | --- | --- | --- | --- | --- | --- | --- | --- | --- | --- | --- | --- | --- | --- | --- | --- | --- | --- | --- | --- | --- | --- | --- |
| **N=1173** |  |  |  |  | **Z1** | **Z2** | **Z3** | **Z4** | **Z5** |  |  | **Z1** | **Z2** | **Z3** | **Z4** | **Z5** |  |  | **BS** | **LT** | **NT** |  |  | **Z1** | **Z2** | **Z3** | **Z4** | **Z5** |  |  | **Z1** | **Z2** | **Z3** | **Z4** | **Z5** |  |  | **BS** | **LT** | **NT** |
| **Patterns** | **N** | **%** | **N** | **%** | **%** | **%** | **%** | **%** | **%** | **N** | **%** | **%** | **%** | **%** | **%** | **%** | **N** | **%** | **%** | **%** | **%** | **N** | **%** | **%** | **%** | **%** | **%** | **%** | **N** | **%** | **%** | **%** | **%** | **%** | **%** | **N** | **%** | **%** | **%** | **%** |
| FS-SH-ZB1 | 351 | 30 | 55 | 16 | 44 | 15 | 13 | 16 | 13 | 160 | 46 | 93 | 3 | 1 | 1 | 1 | 87 | 25 | 40 | 20 | 40 | 13 | 4 | 8 |  | 8 | 69 | 15 | 8 | 2 | 13 |  | 38 | 25 | 25 | 28 | 8 | 18 | 18 | 64 |
| FS-SH-ZB2 | 89 | 8 | 30 | 34 | 33 | 13 |  | 23 | 30 | 11 | 12 |  | 18 | 18 | 45 | 18 | 12 | 13 | 42 | 8 | 50 | 3 | 3 |  |  |  | 33 | 67 | 1 | 1 |  |  | 100 |  |  | 32 | 36 | 38 | 19 | 44 |
| FS-SH-ZB3 | 53 | 5 | 7 | 13 | 14 |  |  | 43 | 43 | 5 | 9 | 20 | 40 |  |  | 40 | 15 | 28 | 73 | 7 | 20 | 4 | 8 | 25 |  |  | 25 | 50 | 2 | 4 |  | 50 | 50 |  |  | 20 | 38 | 30 | 25 | 45 |
| FS-SH-ZB4 | 15 | 1 |  | 0 |  |  |  |  |  | 2 | 13 |  |  |  |  | 100 | 3 | 20 | 33 |  | 67 | 3 | 20 |  |  |  |  | 100 | 1 | 7 |  |  |  | 100 |  | 6 | 40 | 33 | 17 | 50 |
| FS-SH-ZB5 | 11 | 1 | 2 | 18 |  |  |  | 100 |  |  | 0 |  |  |  |  |  | 3 | 27 | 67 | 33 |  | 2 | 18 |  |  |  | 50 | 50 | 1 | 9 | 100 |  |  |  |  | 3 | 27 | 33 |  | 67 |
| FS-MD-ZB1 | 54 | 5 | 10 | 19 | 100 |  |  |  |  | 12 | 22 | 67 | 17 |  |  | 17 | 10 | 19 | 20 | 20 | 60 | 13 | 24 | 31 | 15 | 15 | 23 | 15 | 4 | 7 | 75 |  |  |  | 25 | 5 | 9 | 40 | 20 | 40 |
| FS-MD-ZB2 | 43 | 4 | 2 | 5 | 50 | 50 |  |  |  | 4 | 9 | 25 |  |  | 25 | 50 | 14 | 33 | 36 | 21 | 43 | 8 | 19 |  |  |  | 25 | 75 | 4 | 9 | 25 |  |  | 50 | 25 | 11 | 26 | 27 | 36 | 36 |
| FS-MD-ZB3 | 21 | 2 | 2 | 10 |  |  |  |  | 100 | 3 | 14 | 33 |  |  |  | 67 | 3 | 14 | 67 | 33 |  | 2 | 10 |  |  | 50 | 50 |  | 2 | 10 | 50 |  |  | 50 |  | 9 | 43 | 44 | 11 | 44 |
| FS-MD-ZB4 | 5 | 0 | 1 | 20 | 100 |  |  |  |  |  | 0 |  |  |  |  |  | 3 | 60 | 33 |  | 67 |  | 0 |  |  |  |  |  |  | 0 |  |  |  |  |  | 1 | 20 | 100 |  |  |
| FS-MD-ZB5 | 5 | 0 | 2 | 40 |  |  |  | 50 | 50 |  | 0 |  |  |  |  |  | 1 | 20 | 100 |  |  | 2 | 40 | 50 |  |  | 50 |  |  | 0 |  |  |  |  |  |  | 0 |  |  |  |
| FS-LN-ZB1 | 22 | 2 | 4 | 18 | 75 |  |  | 25 |  |  | 0 |  |  |  |  |  | 1 | 5 |  |  | 100 | 4 | 18 | 50 |  | 25 |  | 25 | 4 | 18 | 75 |  |  | 25 |  | 9 | 41 | 67 | 22 | 11 |
| FS-LN-ZB2 | 36 | 3 | 1 | 3 |  |  |  |  | 100 | 5 | 14 | 40 |  |  | 40 | 20 | 14 | 39 | 36 | 50 | 14 | 5 | 14 | 20 | 20 |  | 20 | 40 | 3 | 8 |  |  |  | 33 | 67 | 8 | 22 | 75 |  | 25 |
| FS-LN-ZB3 | 14 | 1 | 1 | 7 |  |  |  | 100 |  | 1 | 7 | 100 |  |  |  |  | 6 | 43 | 33 | 50 | 17 |  | 0 |  |  |  |  |  |  | 0 |  |  |  |  |  | 6 | 43 | 33 | 17 | 50 |
| FS-LN-ZB4 | 7 | 1 |  | 0 |  |  |  |  |  | 2 | 29 | 50 |  |  | 50 |  |  | 0 |  |  |  | 1 | 14 |  | 100 |  |  |  |  | 0 |  |  |  |  |  | 4 | 57 | 50 | 25 | 25 |
| FS-LN-ZB5 | 7 | 1 |  | 0 |  |  |  |  |  | 1 | 14 | 100 |  |  |  |  | 1 | 14 | 1 |  |  |  | 0 |  |  |  |  |  | 2 | 29 | 100 |  |  |  |  | 3 | 43 | 33 | 33 | 33 |
| SS-SH-ZB1 | 105 | 9 | 14 | 13 | 29 | 7 | 14 |  | 50 | 10 | 10 | 90 |  |  |  | 10 | 52 | 50 | 39 | 14 | 48 | 3 | 3 | 33 | 33 |  | 33 |  | 10 | 10 | 20 | 20 | 10 | 40 | 10 | 16 | 15 | 44 | 13 | 44 |
| SS-SH-ZB2 | 62 | 5 | 7 | 11 | 43 |  | 29 | 14 | 14 | 12 | 19 | 8 | 8 | 17 | 42 | 25 | 15 | 24 | 47 | 27 | 27 | 2 | 3 |  |  | 50 | 50 |  | 1 | 2 |  |  |  | 100 |  | 25 | 40 | 20 | 36 | 44 |
| SS-SH-ZB3 | 30 | 3 | 5 | 17 | 20 | 20 |  | 60 |  |  | 0 |  |  |  |  |  | 5 | 17 | 20 |  | 80 | 1 | 3 |  |  |  |  | 100 | 1 | 3 | 100 |  |  |  |  | 18 | 60 | 17 | 33 | 50 |
| SS-SH-ZB4 | 15 | 1 | 1 | 7 | 100 |  |  |  |  |  | 0 |  |  |  |  |  | 3 | 20 | 67 |  | 33 | 2 | 13 |  |  |  | 50 | 50 | 3 | 20 | 33 |  | 33 |  | 33 | 6 | 40 | 33 | 17 | 50 |
| SS-SH-ZB5 | 10 | 1 |  | 0 |  |  |  |  |  | 2 | 20 |  | 50 |  | 50 |  | 5 | 50 | 40 | 20 | 40 | 1 | 10 |  |  |  | 100 |  |  | 0 |  |  |  |  |  | 2 | 20 | 50 |  | 50 |
| SS-MD-ZB1 | 42 | 4 | 10 | 24 | 50 | 30 |  |  | 20 | 5 | 12 | 60 | 40 |  |  |  | 4 | 10 | 25 | 25 | 50 | 6 | 14 | 83 |  |  | 17 |  | 2 | 5 | 100 |  |  |  |  | 15 | 36 | 27 | 20 | 53 |
| SS-MD-ZB2 | 45 | 4 | 1 | 2 |  |  |  | 100 |  | 6 | 13 | 33 |  |  | 50 | 17 | 15 | 33 | 60 | 13 | 27 | 5 | 11 | 40 |  |  | 40 | 20 | 4 | 9 |  | 25 |  | 25 | 50 | 14 | 31 | 36 | 14 | 50 |
| SS-MD-ZB3 | 21 | 2 | 1 | 5 |  | 100 |  |  |  |  | 0 |  |  |  |  |  | 7 | 33 | 29 |  | 71 |  | 0 |  |  |  |  |  | 1 | 5 |  |  |  | 100 |  | 12 | 57 | 8 | 33 | 58 |
| SS-MD-ZB4 | 9 | 1 | 2 | 22 |  |  | 50 |  | 50 |  | 0 |  |  |  |  |  | 3 | 33 |  | 33 | 67 | 2 | 22 |  | 50 |  | 50 |  |  | 0 |  |  |  |  |  | 2 | 22 | 50 |  | 50 |
| SS-MD-ZB5 | 5 | 0 | 1 | 20 | 100 |  |  |  |  |  | 0 |  |  |  |  |  | 1 | 20 | 100 |  |  | 1 | 20 |  |  |  | 100 |  |  | 0 |  |  |  |  |  | 2 | 40 | 50 |  | 50 |
| SS-LN-ZB1 | 22 | 2 | 7 | 32 | 57 |  |  | 29 | 14 | 3 | 14 | 33 |  | 67 |  |  | 3 | 14 | 33 | 33 | 33 | 7 | 32 | 57 | 29 |  | 14 |  |  | 0 |  |  |  |  |  | 2 | 9 |  | 100 |  |
| SS-LN-ZB2 | 38 | 3 | 6 | 16 | 17 |  |  | 33 | 50 | 6 | 16 | 33 | 17 |  | 33 | 17 | 15 | 39 | 33 | 27 | 40 | 3 | 8 | 67 |  |  |  | 33 | 2 | 5 |  |  |  | 50 | 50 | 6 | 16 | 33 | 17 | 50 |
| SS-LN-ZB3 | 25 | 2 | 3 | 12 | 67 |  |  | 33 |  | 1 | 4 | 100 |  |  |  |  | 8 | 32 | 38 | 38 | 25 | 3 | 12 | 33 |  | 33 |  | 33 | 2 | 8 |  | 50 |  | 50 |  | 8 | 32 | 38 | 25 | 38 |
| SS-LN-ZB4 | 6 | 1 | 1 | 17 |  |  |  |  | 100 |  | 0 |  |  |  |  |  | 4 | 67 |  |  | 100 | 1 | 17 |  | 100 |  |  |  |  | 0 |  |  |  |  |  |  | 0 |  |  |  |
| SS-LN-ZB5 | 5 | 0 |  | 0 |  |  |  |  |  | 1 | 20 |  | 100 |  |  |  | 1 | 20 | 100 |  |  |  | 0 |  |  |  |  |  |  | 0 |  |  |  |  |  | 3 | 60 | 100 |  |  |

*Note.* Points where there was an ACE or double fault are not included. Abbreviations in table 1. RG: Roland Garros; Wi: Wimbledon; US: US Open
