# Supplementary material for: Match analysis and probability of winning a point in elite men’s singles tennis
Source: PLoS One. 2023 Sep 28;18(9):e0286076. doi: 10.1371/journal.pone.0286076 (PMC10538650; doi:10.1371/journal.pone.0286076)
Supplement: S3 Table — (DOCX) [file pone.0286076.s003.docx]

**S3 Table**

*Analysis of different combinations of variables that affect performance (service, rally length, final stroke and point ending) as a function of the court surface.*

| **Patterns** |  |  | **SW** | | **RW** | | **SWW** | | | | | | **SWFE** | | | | | **SWUE** | | | | | **RWW** | | | | | **RWFE** | | | | | **RWUE** | | | | |
| --- | --- | --- | --- | --- | --- | --- | --- | --- | --- | --- | --- | --- | --- | --- | --- | --- | --- | --- | --- | --- | --- | --- | --- | --- | --- | --- | --- | --- | --- | --- | --- | --- | --- | --- | --- | --- | --- |
| **Clay (RG)**  **(N=1660)** | **N** | **%** | **N** | **%** | **n** | **%** | **n** | **%** | **ACE** | **BH** | **FH** | **OT** | **n** | **%** | **BH** | **FH** | **OT** | **n** | **%** | **BH** | **FH** | **OT** | **n** | **%** | **BH** | **FH** | **OT** | **n** | **%** | **BH** | **FH** | **OT** | **n** | **%** | **BH** | **FH** | **OT** |
|  | **1615** |  |  |  |  |  |  |  |  |  |  |  |  |  |  |  |  |  |  |  |  |  |  |  |  |  |  |  |  |  |  |  |  |  |  |  |  |
| FS-SH | 732 | 45 | 560 | 77 | 172 | 24 | 255 | 35 | 38 | 8 | 36 | 17 | 143 | 20 | 37 | 60 | 3 | 162 | 22 | 40 | 56 | 4 | 25 | 3 | 24 | 68 | 8 | 20 | 3 | 25 | 75 |  | 127 | 17 | 28 | 64 | 8 |
| FS-MH | 215 | 13 | 106 | 49 | 109 | 51 | 34 | 16 |  | 24 | 44 | 32 | 25 | 12 | 28 | 56 | 16 | 47 | 22 | 40 | 51 | 9 | 37 | 17 | 24 | 41 | 35 | 19 | 9 | 26 | 68 | 5 | 53 | 25 | 47 | 42 | 11 |
| FS-LH | 134 | 8 | 83 | 62 | 51 | 38 | 31 | 23 |  | 13 | 55 | 32 | 22 | 16 | 41 | 41 | 18 | 30 | 22 | 43 | 37 | 20 | 19 | 14 | 21 | 42 | 37 | 10 | 7 | 20 | 60 | 20 | 22 | 16 | 55 | 41 | 5 |
| SS-SH | 301 | 19 | 163 | 54 | 138 | 46 | 40 | 13 | 3 | 20 | 68 | 10 | 25 | 8 | 36 | 60 | 4 | 98 | 33 | 51 | 48 | 1 | 21 | 7 | 24 | 67 | 10 | 22 | 7 | 18 | 82 |  | 95 | 32 | 38 | 54 | 8 |
| SS-MH | 144 | 9 | 82 | 57 | 62 | 43 | 27 | 19 |  | 30 | 37 | 33 | 19 | 13 | 47 | 53 |  | 36 | 25 | 39 | 50 | 11 | 18 | 13 | 11 | 72 | 17 | 11 | 8 | 36 | 55 | 9 | 33 | 23 | 45 | 42 | 12 |
| SS-LH | 89 | 6 | 51 | 57 | 38 | 43 | 20 | 22 |  | 10 | 35 | 55 | 12 | 13 | 42 | 42 | 17 | 19 | 21 | 58 | 11 | 32 | 13 | 15 | 15 | 54 | 31 | 7 | 8 | 43 | 43 | 14 | 18 | 20 | 50 | 28 | 22 |
| **Grass (WI)**  **(N=1623)** |  |  |  |  |  |  |  |  |  |  |  |  |  |  |  |  |  |  |  |  |  |  |  |  |  |  |  |  |  |  |  |  |  |  |  |  |  |
|  | **1563** |  |  |  |  |  |  |  |  |  |  |  |  |  |  |  |  |  |  |  |  |  |  |  |  |  |  |  |  |  |  |  |  |  |  |  |  |
| FS-SH | 842 | 54 | 669 | 80 | 173 | 21 | 283 | 34 | 44 | 4 | 26 | 26 | 267 | 32 | 47 | 51 | 2 | 119 | 14 | 45 | 44 | 12 | 31 | 4 | 29 | 52 | 19 | 16 | 2 | 63 | 25 | 13 | 126 | 15 | 14 | 61 | 25 |
| FS-MH | 108 | 7 | 59 | 55 | 49 | 45 | 17 | 16 |  | 24 | 12 | 65 | 11 | 10 | 27 | 46 | 27 | 31 | 29 | 42 | 39 | 19 | 18 | 17 | 28 | 22 | 50 | 5 | 5 | 20 | 80 |  | 26 | 24 | 19 | 54 | 27 |
| FS-LH | 53 | 3 | 26 | 49 | 27 | 51 | 7 | 13 |  | 57 | 43 |  | 4 | 8 | 25 | 75 |  | 15 | 28 | 20 | 60 | 20 | 2 | 4 |  | 100 |  | 7 | 13 |  | 100 |  | 18 | 34 | 33 | 44 | 22 |
| SS-SH | 355 | 23 | 194 | 55 | 161 | 45 | 34 | 10 | 3 | 9 | 65 | 24 | 32 | 9 | 47 | 38 | 16 | 128 | 36 | 39 | 55 | 6 | 19 | 5 | 32 | 37 | 32 | 21 | 6 | 33 | 48 | 19 | 121 | 34 | 27 | 59 | 14 |
| SS-MH | 146 | 9 | 74 | 51 | 72 | 49 | 16 | 11 |  | 13 | 50 | 38 | 14 | 10 | 29 | 50 | 21 | 44 | 30 | 21 | 61 | 18 | 26 | 18 | 12 | 42 | 46 | 10 | 7 | 30 | 40 | 30 | 36 | 25 | 28 | 56 | 17 |
| SS-LH | 59 | 4 | 36 | 61 | 23 | 39 | 8 | 14 |  | 13 | 38 | 50 | 7 | 12 | 57 | 43 |  | 21 | 36 | 33 | 52 | 14 | 5 | 8 | 20 | 40 | 40 | 3 | 5 | 33 | 33 | 33 | 15 | 25 | 33 | 40 | 27 |
| **Hard Court (US)**  **(N=1386)** |  |  |  |  |  |  |  |  |  |  |  |  |  |  |  |  |  |  |  |  |  |  |  |  |  |  |  |  |  |  |  |  |  |  |  |  |  |
|  | **1328** |  |  |  |  |  |  |  |  |  |  |  |  |  |  |  |  |  |  |  |  |  |  |  |  |  |  |  |  |  |  |  |  |  |  |  |  |
| FS-SH | 668 | 50 | 541 | 81 | 127 | 19 | 243 | 36 | 61 | 4 | 22 | 12 | 178 | 27 | 43 | 56 | 2 | 120 | 18 | 45 | 50 | 5 | 25 | 4 | 40 | 48 | 12 | 13 | 2 | 31 | 62 | 8 | 89 | 13 | 28 | 63 | 9 |
| FS-MH | 128 | 10 | 67 | 52 | 61 | 48 | 17 | 13 |  | 12 | 29 | 59 | 19 | 15 | 11 | 74 | 16 | 31 | 24 | 48 | 48 | 3 | 25 | 20 | 24 | 56 | 20 | 10 | 8 | 40 | 40 | 20 | 26 | 20 | 54 | 42 | 4 |
| FS-LH | 86 | 6 | 37 | 43 | 49 | 57 | 6 | 7 |  |  | 83 | 17 | 9 | 10 | 22 | 44 | 33 | 22 | 26 | 27 | 73 |  | 10 | 12 | 20 | 50 | 30 | 9 | 10 | 22 | 67 | 11 | 30 | 35 | 40 | 53 | 7 |
| SS-SH | 228 | 17 | 137 | 60 | 91 | 40 | 33 | 14 | 18 | 27 | 33 | 21 | 24 | 11 | 54 | 46 |  | 80 | 35 | 56 | 43 | 1 | 9 | 4 | 33 | 56 | 11 | 15 | 7 | 53 | 47 |  | 67 | 29 | 40 | 54 | 6 |
| SS-MH | 122 | 9 | 56 | 46 | 66 | 54 | 15 | 12 |  | 27 | 20 | 53 | 11 | 9 | 36 | 45 | 18 | 30 | 25 | 43 | 50 | 7 | 14 | 11 | 14 | 43 | 43 | 7 | 6 | 71 | 29 |  | 45 | 37 | 44 | 47 | 9 |
| SS-LH | 96 | 7 | 59 | 62 | 37 | 39 | 17 | 18 |  | 18 | 65 | 18 | 11 | 11 | 36 | 36 | 27 | 31 | 32 | 39 | 55 | 6 | 14 | 15 | 21 | 57 | 21 | 4 | 4 | 50 | 25 | 25 | 19 | 20 | 37 | 58 | 5 |

*Note.* Double fault points are not included. Abbreviations in table 1. RG: Roland Garros; Wi: Wimbledon; US: US Open
