# Supplementary material for: Match analysis and probability of winning a point in elite men’s singles tennis
Source: PLoS One. 2023 Sep 28;18(9):e0286076. doi: 10.1371/journal.pone.0286076 (PMC10538650; doi:10.1371/journal.pone.0286076)
Supplement: Attachment — Submitted filename: PONE-D-22-33800_reviewer MC notes.pdf [file pone.0286076.s004.pdf]

PLOS ONE

Analysis of effectiveness through patterns of play in professional men's tennis

--Manuscript Draft--

**Manuscript Number:**	PONE-D-22-33800
**Article Type:**	Research Article
**Full Title:**	Analysis of effectiveness through patterns of play in professional men's tennis
**Short Title:**	patterns of play in men's tennis
**Corresponding Author:**	Iván Prieto-Lage<br>Universidade de Vigo<br>Pontevedra, SPAIN
**Keywords:**	notational analysis; performance; observational analysis; court surface; match statistics; tennis coaching
**Abstract:**	Notational analysis and new technologies have allowed a better understanding of tennis. Even so, it is necessary to carry out sequential analyses to optimise results in competition. Therefore, the aim of this research was to analyse the effectiveness in patterns of play using the most relevant performance indicators in tennis. A total of 4669 points were analysed on three different surfaces at Grand Slam tournaments in the 2021 season. An observational methodology was used. Different analysis techniques were used to obtain the results: descriptive and chi-square with a significance level of $p<0.05$. First serve effectiveness was 69% on clay, 75% on grass and 75% on hard court. Second serve effectiveness was approximately 55% regardless of the surface. The majority of points, between 65%-77% depending on the court, ended with a short rally (between one and four shots). Approximately 80% of the points played with first serve and short rally were won on serve. With medium rallies, the probability of winning the point is balanced between server and receiver on any surface. The resolution of the point (winner, forced error and unforced error) varies according to surface, serve and rally. The detected patterns of effectiveness can contribute to the optimisation of the training of professional tennis players.
**Order of Authors:**	Iván Prieto-Lage
	Daniel Torres-Santos
	Adrián Paramés-González
	Juan Carlos Argibay-González
	Xoana Reguera-López-de-la-Osa
	Alfonso Gutiérrez-Santiago
**Additional Information:**	
**Question**	**Response**
**Financial Disclosure**<br><br>Enter a financial disclosure statement that describes the sources of funding for the work included in this submission. Review the submission guidelines for detailed requirements. View published research articles from *PLOS ONE* for specific examples.<br><br>This statement is required for submission and **will appear in the published article** if the submission is accepted. Please make	The author(s) received no specific funding for this work.

sure it is accurate.<br><br>**Unfunded studies**<br>Enter: *The author(s) received no specific funding for this work.*<br><br>**Funded studies**<br>Enter a statement with the following details:<br>• Initials of the authors who received each award<br>• Grant numbers awarded to each author<br>• The full name of each funder<br>• URL of each funder website<br>• Did the sponsors or funders play any role in the study design, data collection and analysis, decision to publish, or preparation of the manuscript?<br>• **NO** - Include this sentence at the end of your statement: *The funders had no role in study design, data collection and analysis, decision to publish, or preparation of the manuscript.*<br>• **YES** - Specify the role(s) played.<br><br>* typeset	
**Competing Interests**<br><br>Use the instructions below to enter a competing interest statement for this submission. On behalf of all authors, disclose any competing interests that could be perceived to bias this work—acknowledging all financial support and any other relevant financial or non-financial competing interests.<br><br>This statement is required for submission and **will appear in the published article** if the submission is accepted. Please make sure it is accurate and that any funding sources listed in your Funding Information later in the submission form are also declared in your Financial Disclosure statement.<br><br>View published research articles from *PLOS ONE* for specific examples.	The authors have declared that no competing interests exist.

**NO authors have competing interests**<br><br>Enter: *The authors have declared that no competing interests exist.*<br><br>**Authors with competing interests**<br><br>Enter competing interest details beginning with this statement:<br><br>*I have read the journal's policy and the authors of this manuscript have the following competing interests: [insert competing interests here]*<br><br>* typeset	
**Ethics Statement**<br><br>Enter an ethics statement for this submission. This statement is required if the study involved:<br><br>• Human participants<br>• Human specimens or tissue<br>• Vertebrate animals or cephalopods<br>• Vertebrate embryos or tissues<br>• Field research<br><br>Write "N/A" if the submission does not require an ethics statement.<br><br>General guidance is provided below. Consult the submission guidelines for detailed instructions. **Make sure that all information entered here is included in the Methods section of the manuscript.**	N/A

**Format for specific study types**<br><br>**Human Subject Research (involving human participants and/or tissue)**<br>• Give the name of the institutional review board or ethics committee that approved the study<br>• Include the approval number and/or a statement indicating approval of this research<br>• Indicate the form of consent obtained (written/oral) or the reason that consent was not obtained (e.g. the data were analyzed anonymously)<br><br>**Animal Research (involving vertebrate animals, embryos or tissues)**<br>• Provide the name of the Institutional Animal Care and Use Committee (IACUC) or other relevant ethics board that reviewed the study protocol, and indicate whether they approved this research or granted a formal waiver of ethical approval<br>• Include an approval number if one was obtained<br>• If the study involved *non-human primates*, add *additional details* about animal welfare and steps taken to ameliorate suffering<br>• If anesthesia, euthanasia, or any kind of animal sacrifice is part of the study, include briefly which substances and/or methods were applied<br><br>**Field Research**<br><br>Include the following details if this study involves the collection of plant, animal, or other materials from a natural setting:<br>• Field permit number<br>• Name of the institution or relevant body that granted permission	
**Data Availability**<br><br>Authors are required to make all data underlying the findings described fully available, without restriction, and from the time of publication. PLOS allows rare exceptions to address legal and ethical concerns. See the PLOS Data Policy and FAQ for detailed information.	Yes - all data are fully available without restriction

A Data Availability Statement describing where the data can be found is required at submission. Your answers to this question constitute the Data Availability Statement and **will be published in the article**, if accepted.<br><br>**Important:** Stating 'data available on request from the author' is not sufficient. If your data are only available upon request, select 'No' for the first question and explain your exceptional situation in the text box.<br><br>Do the authors confirm that all data underlying the findings described in their manuscript are fully available without restriction?	
**Describe where the data may be found in full sentences. If you are copying our sample text, replace any instances of XXX with the appropriate details.**<br><br>• If the data are **held or will be held in a public repository**, include URLs, accession numbers or DOIs. If this information will only be available after acceptance, indicate this by ticking the box below. For example: *All XXX files are available from the XXX database (accession number(s) XXX, XXX.).*<br>• If the data are all contained **within the manuscript and/or Supporting Information files**, enter the following: *All relevant data are within the manuscript and its Supporting Information files.*<br>• If neither of these applies but you are able to provide **details of access elsewhere**, with or without limitations, please do so. For example:<br><br>*Data cannot be shared publicly because of [XXX]. Data are available from the XXX Institutional Data Access / Ethics Committee (contact via XXX) for researchers who meet the criteria for access to confidential data.*<br><br>*The data underlying the results presented in the study are available from (include the name of the third party*	All relevant data are within the manuscript and its Supporting Information files.

Powered by Editorial Manager® and ProduXion Manager® from Aries Systems Corporation

and contact information or URL).<br>• This text is appropriate if the data are owned by a third party and authors do not have permission to share the data.<br><br>* typeset	
Additional data availability information:	

Manuscript Click here to access/download;Manuscript;Manuscript - Plos one.docx

Analysis of effectiveness through patterns of play in professional men's tennis

I. Prieto-Lage[a*], Daniel Torres-Santos[a] A. Paramés-González [a], J.C. Argibay-González[a], X. Reguera-López-de-la-Osa[b], A. Gutiérrez-Santiago[a]

[a]Observational Research Group, Faculty of Education and Sport, University of Vigo (Spain)
[b]Education, Physical Activity and Health Research Group (Gies10-DE3), Galicia Sur Health Research Institute (IIS Galicia Sur), SERGAS-UVIGO, (Spain)

*Corresponding Author
PhD Iván Prieto-Lage
Observational Research Group, Faculty of Education and Sport, University of Vigo (Spain)
Mailing Address and Telephone: Facultade de Ciencias da Educación e do Deporte. Campus de A Xunqueira S/N C.P. 36005 Pontevedra (Spain). Telephone: +34 986802053.
Vigo (Spain). E-mail: ivanprieto@uvigo.es ORCID ID 0000-0002-2569-4999

MPT Daniel Torres-Santos
Observational Research Group, Faculty of Education and Sport, University of Vigo (Spain).
E-mail: daniel_torres2000@hotmail.es

PhD Adrián Paramés-González
Observational Research Group, Faculty of Education and Sport, University of Vigo (Spain).
E-mail: aparames@uvigo.es ORCID ID 0000-0003-1874-4270

MPT Juan Carlos Argibay-González
Observational Research Group, Faculty of Education and Sport, University of Vigo (Spain).
E-mail: juan.carlos.argibay@uvigo.es ORCID ID 0000-0002-2036-6814

PhD Xoana Reguera-López-de-la-Osa
Education, Physical Activity and Health Research Group (Gies10-DE3), Galicia Sur Health Research Institute (IIS Galicia Sur), SERGAS-UVIGO, (Spain). E-mail: xreguera@uvigo.es ORCID ID 0000-0002-3190-7188

PhD Alfonso Gutiérrez-Santiago
Observational Research Group, Faculty of Education and Sport, University of Vigo (Spain).
E-mail address: ags@uvigo.es ORCID ID 0000-0001-8071-3833

Analysis of effectiveness through patterns of play in professional men's tennis

Abstract

In Ihis context

Notational analysis and new technologies have allowed a better understanding of tennis. Even so, it is necessary to carry out sequential analyses to optimise results in competition. Therefore, the aim of this research was to analyse the effectiveness in patterns of play using the most relevant performance indicators in tennis. A total of 4669 points were analysed on three different surfaces at Grand Slam tournaments in the 2021 season. An observational methodology was used. Different analysis techniques were used to obtain the results: descriptive and chi-square with a significance level of $p<0.05$. First serve effectiveness was 69% on clay, 75% on grass and 75% on hard court. Second serve effectiveness was approximately 55% regardless of the surface. The majority of points, between 65%-77% depending on the court, ended with a short rally (between one and four shots). Approximately 80% of the points played with first serve and short rally were won on serve. With medium length rallies, the probability of winning the point is balanced between server and receiver on any surface. The point ending The resolution of the point (winner, forced error and unforced error) varies according to surface, serve and rally. The detected patterns of effectiveness can contribute to the optimisation of the training of professional tennis players.

Key words: notational analysis; performance; observational analysis; court surface; match statistics; tennis coaching.

serve type, and rally length

Introduction

Match analysis in tennis is a topic of interest to sport performance researchers, as can be seen in the numerous investigations that have proliferated recently [1–4]. Until the last decade, tennis has not been a sport where notational analysis data has been used for performance improvement, although this has been changing in recent years [1,5].

The introduction of new technologies in this sport is generating a large amount of data on the analysis of the competition. It is now possible to access statistical information of different kinds without difficulty. The Association of Tennis Professionals (ATP), the Women's Tennis Association (WTA) and the International Tennis Federation (ITF) provide statistics on the most relevant competitions through their partnerships with Infosys, SAP and Slam Tracker [6]. This data can help tennis players' teams to optimise training and improve performance in competition. It can also be useful to prepare match strategies and help decision making during the match [5,7].

New analytical techniques have optimised the interpretation of sport data and have led to more meaningful knowledge [8]. One of the most recently employed techniques in sport sciences are predictive models based on tree diagrams, which have proven to be very useful for detecting behavioural patterns in team sports [9], wrestling sports [10,11] and also in racket sports [2], although in the latter case it has not been widely used so far. This data analysis incorporates the so-called performance indicators, which are those variables that have the greatest influence on the final result of the match.

The scientific literature in tennis has corroborated that a fundamental element for the optimisation of results is the ability to decide with which type of stroke, speed or spin to hit the ball at each moment of the match [12], although it also depends on other factors such as weather conditions, the opponent or the type of tournament or round [13,14].

what about court surface, tournament round,, etc?

if we are talking about most researchers, we need to have several refrences here…

them

Need several references here

which is

what is this?

played

zone

need more details on what does this means

In any case, most researchers agree that the serve is the most decisive technical action of the game [3], due to the fact that the server grants the first opportunity to get the point through an ace, an error by the opponent or to obtain a tactical superiority that puts him/her in an advantageous situation during the first shots [12], where it has been shown that most of the points are finished [15]. Another key factor in the match is the type of surface on which the game is played. The ITF classification divides court surfaces into 5 categories (slow, medium slow, medium, medium fast and fast). In the case of slow surfaces (e.g.: clay), the ball will present a higher coefficient of friction, a decreased horizontal speed and an increased bounce height; in the case of fast surfaces (e.g.: grass or hard track) the opposite will be happening [16]. These surfaces change with respect to the tournament [17] and cause points to develop faster or slower [18]. This aspect influences another key performance indicator in the sport: rally length. Researchers have corroborated that most rallies end before five shots, being more accentuated on fast surfaces [19,20]. Another determining factor is the moment of impact with the ball, which is influenced by the place of the previous bounce, the type of stroke made, as well as the place where the shot is directed [21]. These aspects have been studied independently, but not through a sequential analysis that could provide more effective information to improve training and subsequent performance in competition.

Based on the above, the aim of the research was to analyse the effectiveness in the achievement of the point using a system based on patterns of play with the most important performance indicators of tennis.

If this is the goal of the study, the concept of patterns of play should be explained in the introduction. Furthermore, the authors should reference the studies that have previously used this concept in previous research. I am sure there are several studies that have analysed patterns of play in tennis. These papers should be mentioned.
Which are the hypotheses tested in the study? The goal is mentioned, but the authors do not provide any hypotheses to be tested. This is crucial.

Method

Design

The aim of this study is

Study to detect the effectiveness of playing patterns in professional men's tennis in which an observational methodology has been used [22].

The observational design [23] used is nomothetic (all points played in the final rounds of the Grand Slams in 2021 -from quarterfinals-, excluding the Australian Open), follow-up (one season), and unidimensional (there is no concurrence of behaviours).

Sample

I thought we were analysing patterns of play…please clarify…

Since the unit of analysis of this study is the points played in the different matches of the selected tournaments, a total of 4669 points were analysed (1660 at Roland Garros, 1623 at Wimbledon and 1386 at the US Open). All matches from the quarter-finals onwards were analysed, seven per tournament. The study was approved by the Ethics Committee of the Faculty of Education and Sport Science (University of Vigo, Application 02/0320).

Instruments

The observation instrument used for this study is the OBSTENNIS-v3, a system of categories that contemplates in its criteria the different possibilities of play in tennis. It is an adaptation of the OBSTENNIS-v2 [2].

what is this? This is a very vague statement

Has this tool be used before in previous research? if yes, in which one (s), if not, has it been validated?

The OBSTENNIS-v3 (table 1) is made up of seven criteria that form a system of categories that meets the conditions of exhaustiveness and mutual exclusivity. Data registration was performed with LINCE v.1.4 software [24].

Table 1. *OBSTENNIS observation instrument*

CRITERIA	CODE	DESCRIPTION
**SERVICE**	FS	The point is played with first serve.
	SS	The point is played with second serve.
	DF	The server makes a double foul. fault
**RALLY LENGHT**	SH	Short rally (0-4 shots).
	MD	Medium rally (5-8 shots).
	LN	Long rally (9+ shots). court
**BOUNCE ZONE**	SZ	The point ends from the service zone (ace or double fault).
	ZB1 to ZB5	The zone of the surface where the ball bounces before a winner or forced error (in this case the player who wins the point is registered). In the case of an unforced error, the bounce before the error is registered. In the case of a volley or smash, the area where the player's feet are placed is registered.
**END ZONE SHOT**	Z1 to Z5	Zone of the surface where the ball is finally directed (only for winners and forced errors).
	NET	The final shot goes into the net or does not even reach the net.
	LTO	The final shot goes out to the lateral side.
	BSO	The final shot goes out on the baseline.
**WINNER**	SW	The server wins the point.
	RE	The returner wins the point.
**POINT** ENDING **RESOLUTION**	SWW	The server wins with a winner.
	SWFE	The server wins with a forced error.
	SWUE	The server wins with a unforced error.
	RWW	The returner wins a winner.
	RWFE	The returner wins a forced error.
	RWUE	The returner wins a unforced error.
FINISH **RESOLUTION AND FINAL STROKE**	SACE	The server wins with an ACE.
	SWFH	The server wins with a forehand winner.
	SWBH	The server wins with a backhand winner.
	SWOT	The server wins with a winner with another type of stroke (drop shot, smash, volley...).
	SFEFH	The server wins with a forehand by a forced error of the opponent.
	SFEBH	The server wins with a backhand by a forced error of the opponent.
	SFEOT	The server wins with another type of stroke by a forced error of the opponent.
	SUEFH	The server wins with a forehand by an unforced error of the opponent.
	SUEBH	The server wins with a backhand by an unforced error of the opponent.
	SUEOT	The server wins with another type of stroke by an unforced error of the opponent.
	RDF	The returner wins with an ACE
	RWFH	The returner wins with a forehand winner
	RWBH	The returner wins with a backhand winner
	RWOT	The returner wins with a winner with another type of stroke (drop shot, smash, volley...).
	RFEFH	The returner wins with a forehand by a forced error of the opponent.
	RFEBH	The returner wins with a backhand by a forced error of the opponent.
	RFEOT	The returner wins with another type of stroke by a forced error of the opponent.
	RUEFH	The returner wins with a forehand by an unforced error of the opponent.
	RUEBH	The returner wins with a backhand by an unforced error of the opponent.
	RUEOT	The returner wins with another type of stroke by an unforced error of the opponent.

*****Figure 1 here*****

Procedure

Data collection was carried out by recording the matches of three out of the four Grand Slams of the 2021 season (one on each surface).

After adequate training in the use of the instruments, the points were monitored and registered by two expert observers. To ensure rigour in the registration process [25], the quality of the registered data was controlled by calculating intra- and inter-observer agreement using the Kappa coefficient [26] calculated using LINCE software. Both concordances were performed on points that did not belong to the final sample (n=450; 1/10 of the final sample). The intra-observer kappa was 0.93 for the first observer and 0.96 for the second observer. The inter-observer kappa was 0.94.

After the registration of all the points, we obtained an Excel file with the sequentiality

now the authors speak about behaviours…it seems not very clear…patterns, points, behaviours….it should be clarified…

of the behaviours. The versatility of this file allowed us to perform successive transformations for the different analyses [27].

Data Analysis

All statistical analyses were performed using IBM- Statistical Package for the Social Sciences, version 25.0 (IBM-SPSS Inc., Chicago, IL, USA). Statistical significance was assumed for $p<0.05$.

The $\chi 2$ test was used to contrast the differences between the categories of each criterion used (intra-criteria analysis), as well as to compare the differences between the playing surfaces (clay, grass or hard court) of Roland Garros, Wimbledon and US Open (inter-criteria analysis).

now we talk about sequences….what is this? patterns, behaviours, points?

The analysis of the effectiveness of the playing sequences was carried out using the case selection technique combined with a frequency and cross-table analysis.

Results

Descriptive statistical analysis

Table 2 shows the descriptive analysis of the study differentiated by type of surface, as well as the comparative analysis test between surfaces ($\chi2$ inter-criteria).

now back to points…no patterns, behaviours…please clarify

Table 2. *Distribution of points on the different surfaces in the 2021 season and comparative analysis between surfaces ($\chi2$ inter-criteria)*

		**Clay**		**Grass**		**Hard**		**$\chi2$ Inter-criteria**
		n	%	n	%	n	%	
**SERVICE**	DF	45	2,7	60	3,7	58	4,2	$\chi2$=8,174
	FS	1081	65,1	1003	61,8	882	63,6	*p*=.085
	SS	534	32,2	506	34,5	446	32,2	
**RALLY LENGHT**	LN	223	13,4	112	6,9	182	13,1	$\chi2$=74,200
	MD	359	21,6	254	15,7	250	18,0	*p*=.000
	SH	1078	64,9	1257	77,4	954	68,8	
**BOUNCE ZONE**	SZ	141	8,5	187	11,5	213	15,4	$\chi2$=66,987
	ZB1	711	42,8	797	49,1	596	43,0	*p*=.000
	ZB2	441	26,6	339	20,9	313	22,6	
	ZB3	197	11,9	186	11,5	164	11,8	
	ZB4	99	6,0	56	3,5	57	4,1	
	ZB5	71	4,3	58	3,6	43	3,1	
**END ZONE SHOT**	BSO	278	16,7	234	14,4	218	15,7	$\chi2$=87,508
	LTO	229	13,8	155	9,6	121	8,7	*p*=.000
	NET	279	16,8	332	20,5	269	19,4	
	Z1	400	24,1	534	32,9	460	33,2	
	Z2	94	5,7	103	6,3	81	5,8	
	Z3	56	3,4	42	2,6	34	2,5	
	Z4	135	8,1	97	6,0	105	7,6	
	Z5	189	11,4	126	7,8	98	7,1	
**WINNER**	RW	615	37,0	565	34,8	489	35,3	$\chi2$=1,972
	SW	1045	63,0	1058	65,2	897	64,7	*p*=.373
**POINT RESOLUTION**	RWFE	89	5,4	62	3,8	58	4,2	$\chi2$=26,960
	RWUE	393	23,7	402	24,8	334	24,1	*p*=.003
	RWW	133	8,0	101	6,2	97	7,0	
	SWFE	246	14,8	335	20,6	252	18,2	
	SWUE	392	23,6	358	22,1	314	22,7	
	SWW	407	24,5	365	22,5	331	23,9	
**RESOLUTION AND FINAL STROKE**	RDF	38	2,3	60	3,7	58	4,2	$\chi2$=172,046
	RFEBH	23	1,4	22	1,4	25	1,8	*p*=.000
	RFEFH	61	3,7	30	1,8	29	2,1	
	RFEOT	5	,3	10	0,6	5	0,4	
	RUEBH	133	8,0	77	4,7	105	7,6	
	RUEFH	183	11,0	196	12,1	150	10,8	
	RUEOT	40	2,4	69	4,3	20	1,4	
	RWBH	28	1,7	24	1,5	26	1,9	
	RWFH	73	4,4	42	2,6	50	3,6	
	RWOT	31	1,9	35	2,2	21	1,5	
	SACE	99	6,0	125	7,7	155	11,2	
	SFEBH	92	5,5	152	9,4	101	7,3	
	SFEFH	139	8,4	166	10,2	137	9,9	
	SFEOT	16	1,0	17	1,0	14	1,0	
	SUEBH	172	10,4	135	8,3	145	10,5	

SUEFH	193	11,6	181	11,2	157	11,3
SUEOT	26	1,6	42	2,6	12	0,9
SWBH	51	3,1	22	1,4	28	2,0
SWFH	168	10,1	112	6,9	89	6,4
SWOT	89	5,4	106	6,5	59	4,3

Note. Abbreviations in table 1

Statistically significant differences ($p<0.05$) were found between the categories of each of the criteria and on each of the three surfaces analysed (intra-criteria $\chi 2$). In the comparison between surfaces (inter-criteria $\chi 2$ test), statistically significant differences were observed in the type of rally (greater number of medium and long rallies on clay than on grass and hard court), the bounce zone (greater number of actions from zone 2 and fewer from the service on clay compared to the other surfaces), the final direction (among other aspects, fewer finishings with an around the net shot, more shots are sent to the lateral zone and to zone 5 on clay compared to other surfaces), the type of point resolution (on fast courts more points are won on serve due to a forced error by the opponent) and the type of final stroke (for more details see table S1 of the supplementary material).

The results show that first serve accuracy ranged from 65.1-61.8-63.6% depending on the surface (clay, grass or hard). Short rally points predominated (64.9-77.4-68.8%) although they were more frequent on fast surfaces.

Nearly half of the points analysed on the three surfaces ended after a bounce previous to the final shot into zone 1 (42.8-49.1-43%) and ended with a winner or forced error in zone 1 (24.1-32.9-33.2%). The points obtained (with a winner or forced error by the opponent) in zone 5 also stood out, especially on clay (11.4%). The surface where most points were won after the return was clay (37%), compared to 34.8% on grass and 35.3% on hard court. Both, serving and return, it is on clay where more winners were obtained (24.5% and 8% respectively; on grass 22.5% and 6.2% and on hard court 23.9% and 7%), although it is also the surface where

fewer points were obtained by forced error serving (14.8% compared to 20.6% and 18.2%). An even amount of points won by returning (23.7-24.8-24.1%) and serving (23.6-22.1-22.7%) by unforced error was registered on all three surfaces. The surface with the most aces was hard court (11.2%) and the least on clay (6%). Unforced errors on the return had similar percentages after hitting with the forehand and backhand; however, when serving, they were much more frequent with the forehand. Winners are more frequent with the forehand, both serving and returning, and were considerably more frequent when serving.

Sequential analysis

Do you mean analysis of patterns?

Figure 2 shows a comparative analysis of the effectiveness of the point based on service, rally length and surface type.

Figure 2 here

The effectiveness with the first serve was 69% on clay, 75% on grass and 75% on hard. Second serve effectiveness was 55% on clay, 54% on grass and 57% on hard, a decrease in effectiveness of 14-21-18% respectively.

Statistically significant differences were observed between surfaces in the first service-long rally sequence ($\chi 2$=8.052; sig.=0.018). On both, grass and hard court, the returner was more effective in this type of pattern of play, but not on clay.

Figure 3 and figure 4 show an analysis, according to the type of surface and pattern of play (service and rally), of the distribution of points with respect to the type of resolution (winners, forced errors or unforced errors).

*****Figure 3 here*****

*****Figure 4 here*****

Relevant differences between surfaces were found in the first service −short rally pattern ($\chi2$=8.052; sig.=0.018)− and in the first service −long rally pattern ($\chi2$=26.106; sig.=0.004). In the first of the aforementioned sequences, a greater number of points finished by unforced errors were registered on clay than on the rest of the surfaces, both on serve and on the rest. Likewise, the number of forced errors (made by the returner) obtained when serving was higher on grass than on the rest of the surfaces. In the second sequence, the number of unforced errors in points won on return was far higher on grass and hard than on clay. More winners were also observed on clay than on grass and hard (in service as well as in return points).

Supplementary Material Table S2 includes an analysis of the distribution of the points according to the previously studied patterns of play (surface type, service and rally), but also including the bounce zone previous to the final stroke (ZB1-ZB5), as well as the final direction zone of the last stroke (Z1-Z5 or BS, LT or NT).

The most frequent pattern of play found was the first serve, short rally and final shot after a bounce in zone 1, representing 25-35-30% of the total depending on the surface (clay, grass and hard court). In this situation, only 14% of the points were won on the return (the value is the same on all three surfaces). When serving, it is on clay where more points were won by winners (23%) and by unforced errors by the opponent (33%). The percentage of winners to zone 1 (41%) and zone 5 (28%) stands out in comparison with the others. Unforced errors are preceded by a shot that goes out over the baseline (52%). On grass, forced errors (48%) were more frequent and were mostly directed to zone 1 (88%). This was also the case with winners (52%), but those directed to zone 2 (15%) and zone 5 (16%) also represent a significant

proportion of the total. The points won by serving through an unforced error by the opponent are usually sent to the net (59%). Most of the points won by the returner are by unforced error with a shot to the net (59%). On hard court, the data was similar to that on grass, with a high number of forced errors (46%) sent to zone 1 (93%). Winners (16%) were mostly directed to zone 1 (44%) but there were shots sent to all other zones (around 15%).

In Supplementary Material Table S3 an analysis of patterns of play was carried out taking into account the type of surface, the service and the rally as a function of the way of resolution and the final stroke (forehand, backhand or other stroke).

On clay, winners when the player served were more frequent with the forehand than with the backhand in all the possible match situations studied, something that did not occur on grass or hard court. Returning, winners were predominantly with the forehand on clay and hard court, but on grass, many points were resolved with another type of stroke (probably volleys). Unforced error points won on serve were very even on clay with first serves (regardless of the type of rally) and second serves in short rallies. On grass and hard courts, this balance was only evident with first serves in short and medium rallies. In all other cases, forehand errors predominated, except in short rallies in second serves on hard courts.

The points won by unforced errors on grass when the player was returning was linked to a forehand (occurred in all possible solutions). On clay it was the same in short rallies (both first and second serves), there was a balance between forehand and backhand errors in medium rallies and in long rallies the error was preceded by a backhand stroke. On the hard surface, there was a balance of forehand and backhand errors in almost all the situations analysed, except for the first serve and short rallies, where the errors were clearly caused by a forehand.

Discussion

This study seeks to expand on the limited information on the effectiveness of different

now patterns…what about behaviours or sequences…

patterns of play by surface (clay, grass or hard) by analysing their impact on the final rounds of three Grand Slam events on the men's tour in 2021, taking into account the most important performance indicators in tennis.

this is a new concept here…not sure if it had been mentioned before. If not, may be it's too late. If yes, then it should be explained and the adequate references cited in the introduction.

The results obtained on first serve accuracy are similar to the results found in other research [3,28], where it was registered between 58-64% on clay, 65% on grass and 63-65% on hard court (65.1-61.8-63.6% in our study respectively). The first serve effectiveness rate in our study was 69.2%-75%-75% depending on the surface. Other researchers [3,18,28] evidenced an effectiveness ranging from 69-75% on clay, 75-79% on grass and 70-78% on hard court. According to the researchers cited above, the effectiveness of the second serve fluctuates between 51-56%, 53-58% and 48-55% depending on the surface, values also close to the 54.6%-54%-56% obtained in our case in the 2021 season. These results are consistent with the friction coefficient and coefficient of restitution data that place grass as the surface with the highest speed, ahead of hard court and clay [29]. It is confirmed that the first serve is fundamental to win matches in this sport, especially on fast surfaces [12,16,30,31]as the loss of efficiency with second serves is notable, being more accentuated on fast courts. Considering that playing second serves generates a probability of winning the point of around 55%, raising the percentage of first serves should be a key issue in training, as it would increase the probability of success by up to 14-21% depending on the surface.

Most of the points, although there are differences between surfaces, were played with a short rally (64.9% on clay, 77.4% on grass and 68.8% on hard). The literature has contrasted that, over the last few years, the frequency of this type of rally has increased on all surfaces due

to more aggressive playing strategies by players, where they put pressure on the opponent from the very beginning [32–34]. Furthermore, it has also been shown that players who dominated short rallies (0-4 strokes) won the match in 9 out of 10 cases, both on clay and grass [15,20], hence tactical trainings should try to optimise this aspect of the game. The best strategy for the player serving with a first serve and short rally was to look for a winner, as 34-36% of the cases obtained the point later on either surface. In addition, especially on grass, a high percentage of forced errors by the opponent was registered (32%), dropping to 27% on hard and 20% on clay. The best return strategy was to look for an unforced error from the opponent, regardless of the surface, although this was more effective on clay (17%) than on grass (15%) or hard court (13%). Return winners after the first serve were rather scarce in short rallies (3-4%). With this information, and considering that the returner is probably always at a tactical disadvantage [12], training to work on the defence of the serve should be aimed at trying to put the server in a situation of discomfort, rather than trying to find a winner, at least until after four rallies. Hitting the ball to zone 3 (baseline) and zone 4 (backhand on the right-handed player) were the most effective, confirming that the search for the opponent's error occurs more frequently on the backhand stroke [21].

Medium rally points represented 21.6-15.7-18.0% in this study depending on the surface. According to one study [20], dominance on these points determines the winner of a clay court match by 65% and 69% on grass (approximately 25% less than dominance on short rallies). With the first serve, the tactic of looking for a winner on either surface was not more effective, but rather the search for an unforced error from the opponent. When returning the serve the same happened. The points won by unforced error were very balanced between server and returner, so it seems clear that, at a strategic level, the server is not interested in reaching this type of rally [35]. This highlights the importance of having an effective service to end the

point as soon as possible and thus improve the probability of success at the point [12,30,35,36]. With the second serve, the statistics do not vary much with respect to the first serve, except that the points won by unforced errors on hard courts increase (+16.6%). This would suggest that the effect of the serve is diluted after the fifth shot, something that has already been suggested in the literature [35] On clay, with this rally and second serve, the tactic of looking for a winner (winner or forced error) was more effective than looking for the unforced error. In the return game, the opposite was true. On grass it was more effective to look for the unforced error both on serve and return. On hard courts it was much more effective to look for the unforced error, especially in the return.

Long rally points were few in general, although this fact was more pronounced on grass, where not even 7% were observed. Although it was not investigated whether these points were important points (e.g.: breakpoints), the literature has pointed out that dominance on these types of points is linked to match success in 66% of cases on clay and 61% on grass [15], quite similar to that of medium rallies, so they must also have their importance in training, both from a physical, psychological and strategic point of view. In fact, there is a curious fact, at least on fast surfaces. The server won fewer points after a first serve than the returner, but significantly increased his effectiveness with a second serve. The explanation for this phenomenon is complex, but it may possibly be linked to aspects of concentration, due to starting from a disadvantageous situation (second serve).

Practical application

Throughout this article, different guidelines have been established for patterns of effectiveness depending on the service, surface and rally. In any case, we recommend that coaches make use of the different tables and figures in the manuscript, as well as the supplementary material to obtain more specific data, where an analysis is also made according

to the bounce zone prior to the last shot, type of final stroke and direction of the final shot, all differentiated by surface, type of service and rally length.

Limitations and future perspectives

In this study we have only analysed matches from the Grand Slam quarter-finals onwards, so previous rounds have not been taken into account. The direction of the serve (wide, body or T zone) and its speed have not been considered, aspects that could be interesting to address in future research.

Bearing in mind that most of the points end in short rallies (0-4 shots), in the future studies should be carried out linked to the exact number of shots in the point, focusing on this type of rally, in order to establish more specific patterns of effectiveness, taking into account all the movements and strokes made by the players.

Conclusions

The first serve is essential for the effectiveness on all types of rally and surfaces. Starting the point on the second serve decreases the effectiveness by 14.6% (clay), 21% (grass) or 17% (hard court) compared to the first serve. The probability of winning the point with a first serve and medium rally decreases by 25-29% depending on the surface compared to finishing with a short rally. On grass only 7% of points end with a long rally (13% on the other two surfaces). The most repeated pattern of play and the most effective for the server was the start of the point with a first serve with a final shot after a bounce in the service zone (zone 1) after a rally of less than five shots. This generated an unforced error in the opponent (on clay) or a forced error (on grass and hard court).

References

1. Fitzpatrick A, Stone JA, Choppin S, Kelley J. Investigating the most important aspect of elite grass court tennis: Short points. Int J Sport Sci Coach. 2021. doi:10.1177/1747954121999593

2. Prieto-Lage I, Paramés-González A, Argibay-González JC, Reguera-López-de-la-Osa X, Ordóñez-Álvarez S, Gutiérrez-Santiago A. Match Analysis in Women's Tennis on Clay, Grass and Hard Courts. Int J Environ Res Public Health. 2022;19: 7955. doi:10.3390/ijerph19137955

3. Fernández-García ÁI, Blanca-Torres JC, Hernández-García R, Torres-Luque G. Analysis of the statistical variables related to the service in high performance male tennis in junior and absolute category. Cultura_Ciencia_Deporte. 2019;14: 289–295.

4. Cui Y, Gómez MÁ, Gonçalves B, Sampaio J. Performance profiles of professional female tennis players in grand slams. PLoS One. 2018. doi:10.1371/journal.pone.0200591

5. Reid M, Morgan S, Whiteside D. Matchplay characteristics of Grand Slam tennis: implications for training and conditioning. J Sports Sci. 2016;34: 1791–1798. doi:10.1080/02640414.2016.1139161

6. Torres-Luque G, Blanca-Torres JC, Cabello-Manrique D, Fernández-García AI. Serve profile of male and female professional tennis players at the 2015 Roland Garros Grand Slam tournament. Ger J Exerc Sport Res. 2019;49: 319–324. doi:10.1007/s12662-019-00615-z

7. Reid M, McMurtrie D, Crespo M. Title: The relationship between match statistics and top 100 ranking in professional men's tennis. Int J Perform Anal Sport. 2010;10: 131–138. doi:10.1080/24748668.2010.11868509

8. Ofoghi B, Zeleznikow J, MacMahon C, Raab M. Data Mining in Elite Sports: A Review and a Framework. Meas Phys Educ Exerc Sci. 2013;17: 171–186. doi:10.1080/1091367X.2013.805137

9. Prieto-Lage I, Louzao-Neira I, Argibay-González JC, Gutiérrez-Santiago A. Injury patterns of professional footballers in the Spanish first division during the 2017–2018 seasons. Physiol Behav. 2020;224: 113052. doi:10.1016/j.physbeh.2020.113052

10. Camerino O, Prieto I, Lapresa D, Gutiérrez-Santiago A, Hileno R. T-pattern detection in combat sports. Rev Psicol del Deport. 2014;23: 147–155.

11. Gutiérrez-Santiago A, Gentico-Merino LA, Prieto-Lage I. Detection of the technical-tactical pattern of the scoring actions in judo in the men's category of –73 kg. Int J Perform Anal Sport. 2019;19: 778–793. doi:10.1080/24748668.2019.1655934

12. Gillet E, Leroy D, Thouvarecq R, Stein JF. A notational analysis of elite tennis serve and serve-return strategies on slow surface. J strength Cond Res. 2009;23: 532–539. doi:10.1519/JSC.0B013E31818EFE29

13. O'Donoghue PG. The most important points in grand slam singles tennis. Res Q Exerc Sport. 2001;72: 125–131. Available: http://www.tandfonline.com/doi/abs/10.1080/02701367.2001.10608942

14. Cui Y, Gómez MÁ, Gonçalves B, Liu H, Sampaio J. Effects of experience and relative quality in tennis match performance during four Grand Slams. Int J Perform Anal Sport. 2017;17: 1–19. doi:10.1080/24748668.2017.1399325

15. Fitzpatrick A, Stone JA, Choppin S, Kelley J. Important performance characteristics in elite clay and grass court tennis match-play. Int J Perform Anal Sport. 2019;19: 942–952. doi:10.1080/24748668.2019.1685804

16. Vaverka F, Nykodym J, Hendl J, Zhanel J, Zahradnik D. Association between serve speed and court surface in tennis. Int J Perform Anal Sport. 2018;18: 262–272. doi:10.1080/24748668.2018.1467995

17. Bermejo J. Análisis Estructural Del Golpeo En Tenis. Una Aproximación Desde La Biomecánica. Mot Eur J Hum Mov. 2013;31: 111–133.

18. Doğan İ, Revan S, Arikan Ş. Analysis of tennis competitions on different court surface. Turkish J Sport Exerc. 2021;23: 60–66. doi:10.15314/tsed.895726

19. Torres-Luque G., Sánchez-Pay A., Fernández-García ÁI., Palao JM. Characteristics of Temporal Structure in Tennis. A review. J Sport Heal Res. 2014;6: 117–128.

20. Fitzpatrick A, Stone JA, Choppin S, Kelley J. A simple new method for identifying performance characteristics associated with success in elite tennis. Int J Sports Sci Coach. 2019;14: 43–50. doi:10.1177/1747954118809089

21. Fernández-García ÁI, Juan-Campos JM, Giménez-Egido JM. Analysis of Technical-Tactical Variables Related to the Last Stroke of the Point in High Performance Tennis. Jump. 2020; 48–56. doi:10.17561/jump.n2.5

22. Anguera MT, Blanco-Villaseñor A, Losada JL, Portell M. Guidelines for designing and conducting a study that applies observational methodology. Anu Psicol. 2018;48: 9–17. doi:10.1016/j.anpsic.2018.02.001

23. Anguera MT, Blanco-Villaseñor A, Hernández-Mendo A, Losada-López JL. Observational designs: Their suitability and application in sports psychology. Cuad Psicol del Deport. 2011;11: 63–76.

24. Gabin B, Camerino O, Anguera MT, Castañer M. Lince: Multiplatform Sport Analysis Software. Procedia - Soc Behav Sci. 2012;46: 4692–4694. doi:10.1016/j.sbspro.2012.06.320

25. Blanco-Villaseñor A, Anguera MT. Evaluación de la calidad en el registro del comportamiento: Aplicación a deportes de equipo. In: Oñate E, García-Sicilia F, Ramallo L, editors. Métodos Numéricos en Ciencias Sociales. Barcelona: Centro Internacional de Métodos Numéricos en Ingeniería; 2000. pp. 30–48.

26. Cohen J. Weighted kappa: Nominal scale agreement with provision for scaled disagreement of partial credit. Psychol Bull. 1968;70: 213–220. doi:10.1037/h0026256

27. Prieto-Lage I, Prieto MA, Curran TP, Gutiérrez-Santiago A. An Accurate and Rapid System to Identify Play Patterns in Tennis Using Video Recording Material: Break Point Situations as a Case Study. J Hum Kinet. 2018;62: 199–212. doi:10.1515/hukin-2017-0170

28. Sánchez-Pay A, Palao JM, Torres-Luque G, Sanz-Rivas D. Differences in set statistics between wheelchair and conventional tennis on different types of surfaces and by

gender. Int J Perform Anal Sport. 2015;15: 1177–1188. doi:10.1080/24748668.2015.11868860

29. Brody H. Bounce of a tennis ball. J Sci Med Sport. 2003;6: 113–119. doi:10.1016/S1440-2440(03)80014-2

30. Mecheri S, Rioult F, Mantel B, Kauffmann F, Benguigui N. The serve impact in tennis: First large-scale study of big Hawk-Eye data. Stat Anal Data Min. 2016;9: 310–325. doi:10.1002/sam.11316

31. Klaus A, Bradshaw R, Young W, O'Brien B, Zois J. Success in national level junior tennis: Tactical perspectives. Int J Sports Sci Coach. 2017;12: 618–622. doi:10.1177/1747954117727792

32. Katić R, Milat S, Zagorac N, Durović N. Impact of game elements on tennis match outcome in Wimbledon and Roland Garros 2009. Coll Antropol. 2011;35: 341–6. Available: http://www.ncbi.nlm.nih.gov/pubmed/21755700

33. Stare M, Žibrat U, Filipčič A. Stroke Effectiveness in Professional and Junior Tennis. Kinesiol Slov. 2015;21: 39–50.

34. Filipčič T, Filipčič A, Berendijaš T. Comparison of game characteristics of male and female tennis players at Roland Garros 2005. Acta Gymnica. 2008;38: 21–28.

35. O'Donoghue PG, Brown E. The Importance of Service in Grand Slam Singles Tennis. Int J Perform Anal Sport. 2008;8: 70–78. doi:10.1080/24748668.2008.11868449

36. O'Donoghue PG, Brown E. Sequences of service points and the misperception of momentum in elite tennis. Int J Perform Anal Sport. 2009;9: 113–127. doi:10.1080/24748668.2009.11868468

Figure 1

Click here to access/download;Figure;Figure 1.tif

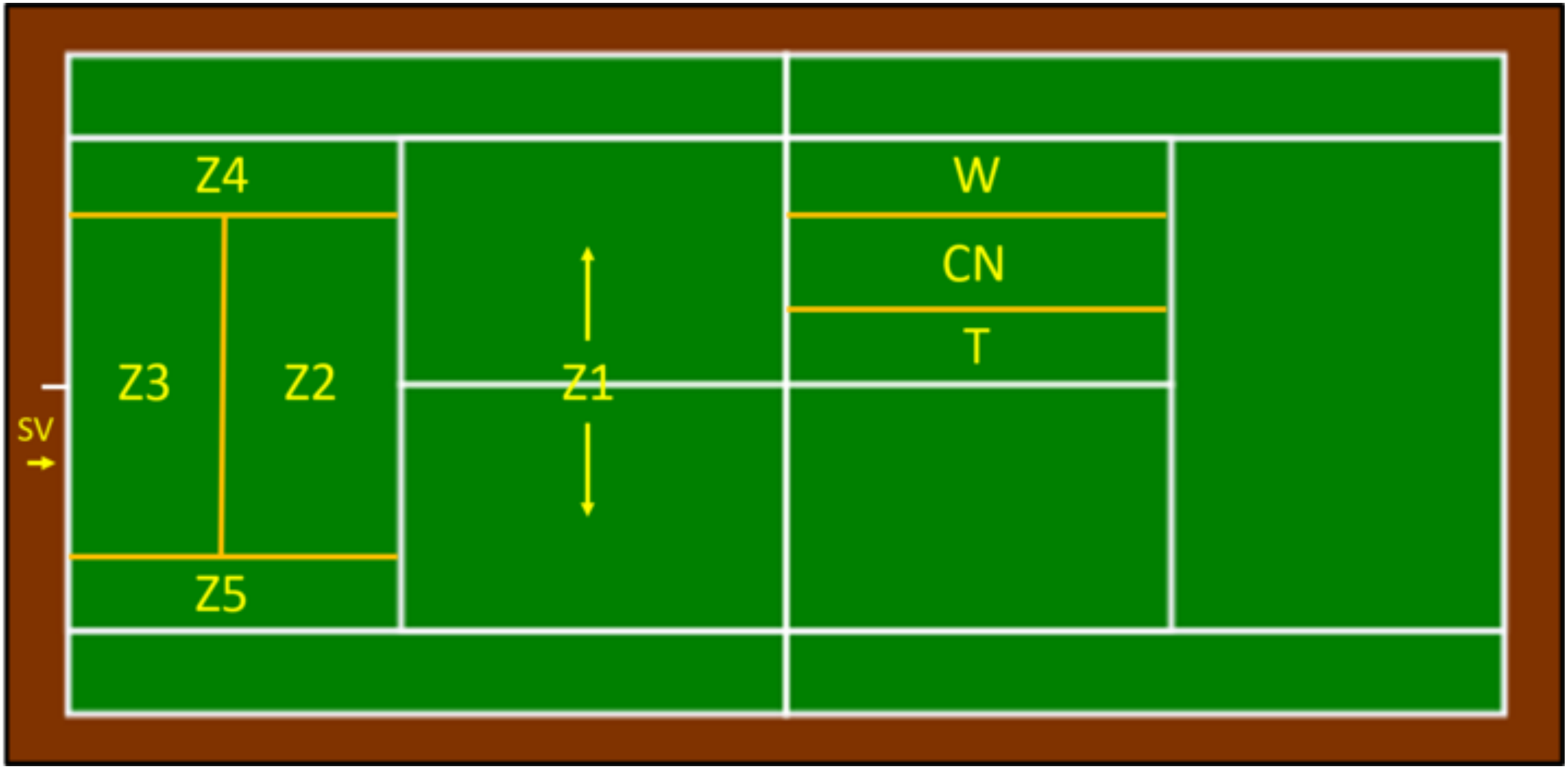

How were these zones decided?
Have they been used in previous studies?
Which are their references?
Have they been invented by the authors?

Figure 2

Click here to access/download;Figure;Figure 2.tif

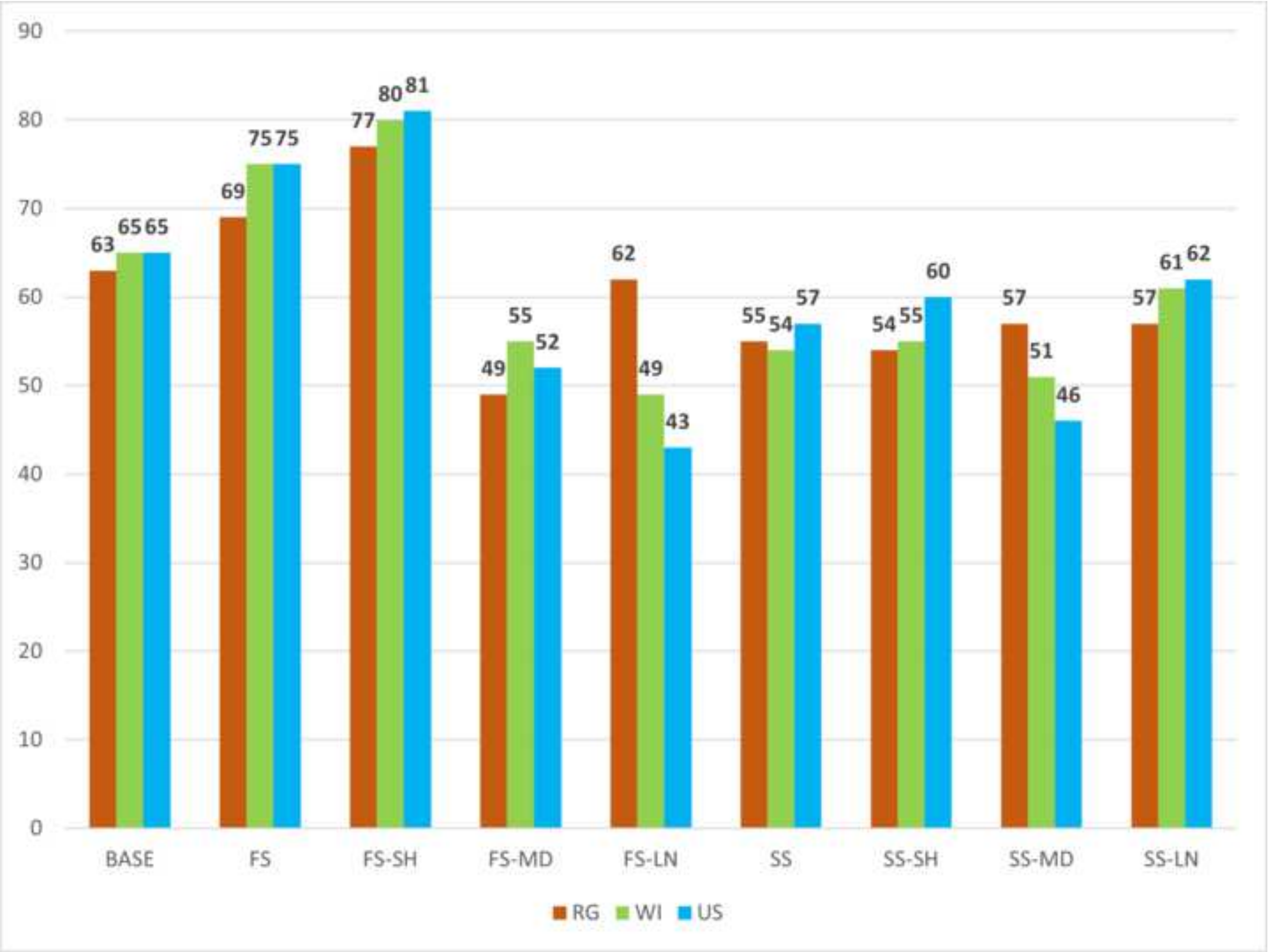

Figure 3

Click here to access/download;Figure;Figure 3.tif

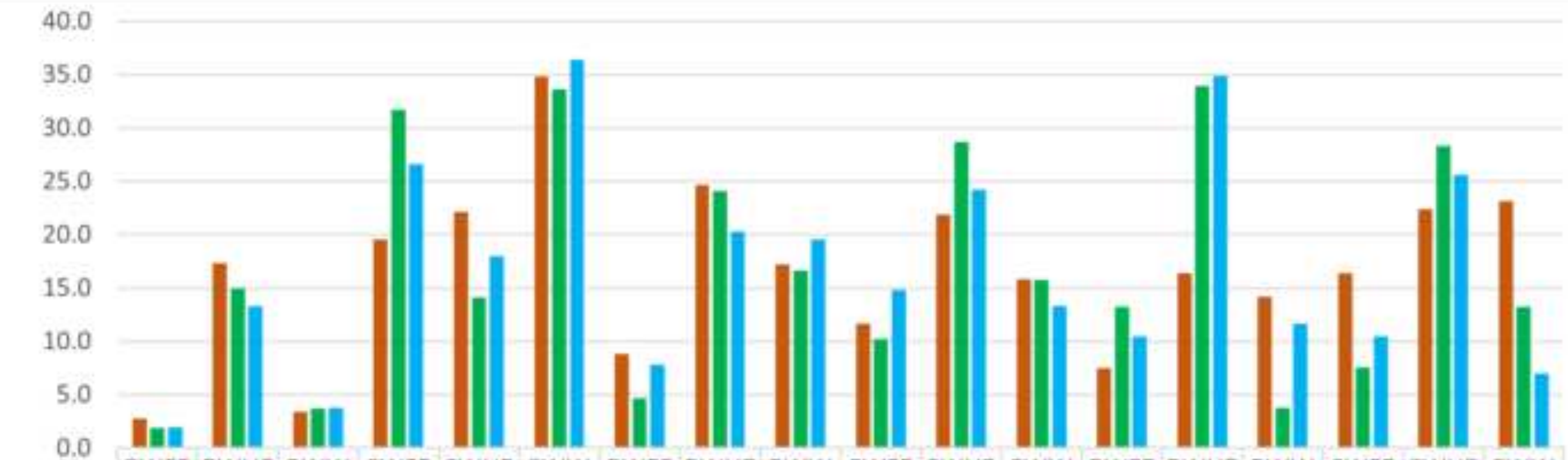

	FS-SH						FS-MD						FS-LN					
	RWFE	RWUE	RWW	SWFE	SWUE	SWW	RWFE	RWUE	RWW	SWFE	SWUE	SWW	RWFE	RWUE	RWW	SWFE	SWUE	SWW
CLAY	2.7	17.3	3.4	19.5	22.1	34.8	8.8	24.7	17.2	11.6	21.9	15.8	7.5	16.4	14.2	16.4	22.4	23.1
GRASS	1.9	15.0	3.7	31.7	14.1	33.6	4.6	24.1	16.7	10.2	28.7	15.7	13.2	34.0	3.8	7.5	28.3	13.2
HARD	1.9	13.3	3.7	26.6	18.0	36.4	7.8	20.3	19.5	14.8	24.2	13.3	10.5	34.9	11.6	10.5	25.6	7.0

Figure 4

Click here to access/download;Figure;Figure 4.tif

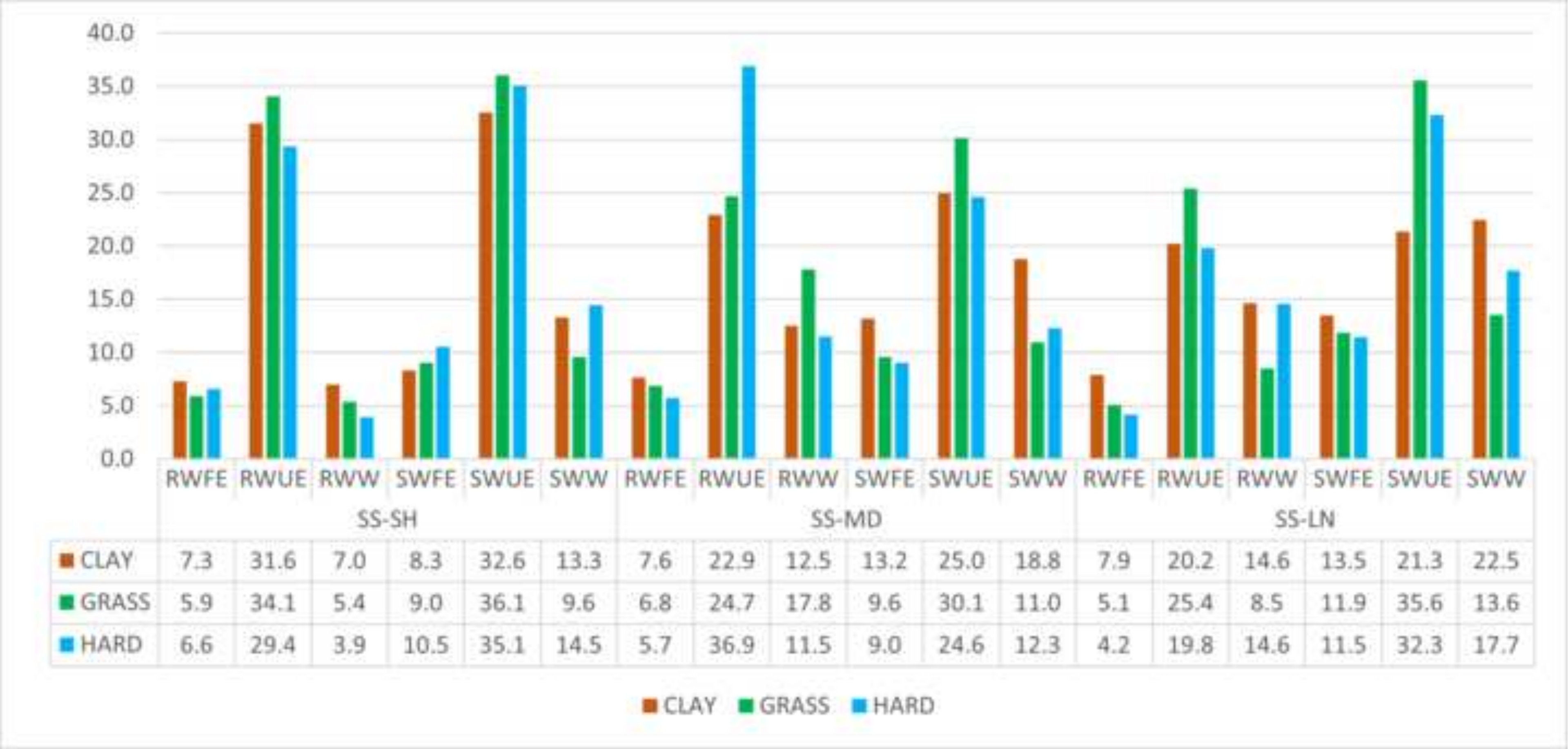

	SS-SH						SS-MD						SS-LN					
	RWFE	RWUE	RWW	SWFE	SWUE	SWW	RWFE	RWUE	RWW	SWFE	SWUE	SWW	RWFE	RWUE	RWW	SWFE	SWUE	SWW
CLAY	7.3	31.6	7.0	8.3	32.6	13.3	7.6	22.9	12.5	13.2	25.0	18.8	7.9	20.2	14.6	13.5	21.3	22.5
GRASS	5.9	34.1	5.4	9.0	36.1	9.6	6.8	24.7	17.8	9.6	30.1	11.0	5.1	25.4	8.5	11.9	35.6	13.6
HARD	6.6	29.4	3.9	10.5	35.1	14.5	5.7	36.9	11.5	9.0	24.6	12.3	4.2	19.8	14.6	11.5	32.3	17.7

Click here to access/download
Supporting Information
Suplementary material Table S1-S2-S3.docx
