# Supplementary material for: Match analysis and probability of winning a point in elite men’s singles tennis
Source: PLoS One. 2023 Sep 28;18(9):e0286076. doi: 10.1371/journal.pone.0286076 (PMC10538650; doi:10.1371/journal.pone.0286076)
Supplement: Attachment — Submitted filename: Response to reviewers.docx [file pone.0286076.s005.docx]

**Reviewer #1:**

**Comment 1.** Initial comments

The reviewer would like to congratulate the authors for conducting research in tennis. More specifically, for studying the strategy of the game at the high-performance level.
The purpose of this review is to share with the authors several comments that will help to improve the quality of the manuscript since, in the views of the reviewer, there are some relevant aspects that need to be clarified, changed, referenced and deleted.
To assist in the review, the reviewer has enclosed the original pdf of the manuscript with his suggestions and comments. By doing this, the authors will find it easier to follow the recommendations.

**Response Comment 1**

Dear Miguel Crespo. First of all, we would like to thank you for the comments you have made on the document. We are sure that they will greatly improve the quality of the manuscript. The pdf document was of great help, thank you.

You have done an excellent job of review, for which we are deeply grateful. As you well know, tennis is a complex sport to analyse, but we wanted to make an effort to provide scientific information about our sport. We have tried to take all your suggestions into account. In any case, we are open to improve more aspects if you think so.

**Comment 2.** Structure of the paper

No comments regarding the structure of the manuscript.

**Response Comment 2**

Thank you.

**Comment 3.** Language

The reviewer suggests that the language is double checked by an English native expert. More importantly, it has to be someone that is familiar with the game of tennis, since some of the words and constructions used in the text are not completely accurate.

**Response Comment 3**

We have revised the document based on your comments and have asked a translator to revise the text. Because of the technical language of tennis, it is true that there were some expressions that were not correct.

**Comment 4.** Introduction

The authors mention the concept of patterns of play but this concept is not defined in the manuscript. Throughout the document there seems to be a confusion between shots, patterns, behaviours, sequences, etc. This has to be clarified by the authors using the adequate references.

**Response Comment 4a**

We have reviewed the terminology throughout the document. We wanted to use these terms as synonyms in order not to repeat the same words. In the revised version, the article will be treated as the analysis of the possibility of winning the point based on different performance indicators (serve, rally, type of stroke...). We will use "combination of performance indicators" throughout the paper to establish the relationship between variables, e.g. first serve - short rally.

The authors state the objective of the paper but they do not include any hypothesis that they would like to test. This is a very important aspect that also needs to be addressed. Hypotheses have to be included and then, the results and the discussion should refer to them.

**Response Comment 4b**

In observational research, it is not common to hypothesise, but to work with objectives. This is more common in experimental work. In any case, the text has been changed considerably and differentiated sub-sections have been created in the results that are clearly related to the stated objectives.

On the website http://www.observesport.com/# (in the publications section) you can see that in the different articles related to sport and observational methodology there is no reference to hypotheses. This research group is the most prolific observational methodology group in Spain. Our research group http://www.iobserving.com/ (which actively collaborates with it) does not usually raise hypotheses in its studies with observational methodology.

In any case, we are open to the possibility of making improvements or changes to this revised version if it seems appropriate to you.

**Comment 5.** Methodology

The authors use an observational methodology based on an instrument that it is not indicated if it has been used in previous research nor if it has been validated. Again, this has to be clarified to ensure that the tool used is adequate for the purposes of the study.

Furthermore, they use a division of the tennis court in zones that, again, it is not indicated if it has been used in previous studies, nor if it has been validated. The reviewer is not sure where they have taken the references to divide the court in those zones.

Finally, with the technological advances available nowadays, the notational analysis is mostly conducted using other types of methodologies provided by the statistics and computer programmes available.

**Response Comment 5**

We have modified the instrument section in response to your comment. The observation instrument for this study has been validated. In addition, it is based on another validated instrument that used this court zone division. All this information now appears in the text.

We agree that we could have a debate about the division of the court zone. In fact, we are currently working on another type of research where we use a different division. In any case, tennis experts considered this division appropriate for this study for these purposes.

**Comment 6.** Results and discussion

The reviewer feels that the results should be structured based around the hypotheses set. If this is not the case, they will not be clear enough.

**Response Comment 6**

We have changed the structure of the paper in this revised version. Although we have not worked with hypotheses, the objectives have been more specific. In addition, it is reflected throughout the document that, among the performance factors analysed, we have focused on the analysis of the combination of service and rally length. The discussion focuses on these two indicators, contrasting the differences depending on the surface (in each paragraph we focus on the analysis of the serve or a type of rally).

**Comment 7.** Conclusion.

They seem adequate for the goal of the paper. However, the reviewer feels that the limitations and the practical applications should be part of the conclusion and not be included in the discussion.

**Response Comment 7**

Thank you for your suggestion. We have added the limitations and practical implications in the conclusion. Other journals request that these subsections be at the end of the discussion. This was the reason for including the limitations and practical implications in the discussion.

**Comment 8.** Final recommendation.

Due to the comments made above, the reviewer feels that the manuscript needs some major changes that will substantially improve its quality. Finally, the reviewer would like to thank again the authors for their passion and effort in producing this manuscript in tennis.

Miguel Crespo, PhD.

**Response Comment 8**

Thank you very much for all your comments and contributions. They have certainly helped to improve this research. Thank you very much also for your congratulations for doing research in this sport. It is certainly important to us.

**Reviewer #2:**

The report provides a retrospective analysis of categorical data of elite male tennis players in the final rounds of the most prestigious tournaments. The report suffers from unclear writing that makes it difficult for even motivated, tennis-interested readers to follow. The specific comments to the authors outline weaknesses that should be addressed.

**Response 1:**

We have made an effort to improve the document. We have followed all your suggestions for improvement. We have simplified the text to make it easier to read and understand. In addition, an expert in English and sport has made a revision. We are sorry for any language-related errors. We would also like to thank you for your review. It has been very good and we consider it appropriate. We believe that the document has been substantially improved thanks to your comments.

**Page-Line**

**ABSTRACT**

2-4 . . . understanding of tactical actions in tennis. In particular, sequential analysis is needed to understand the relationships between actions in competitions.
**Response 2:**

We have modified the text according to your comment. Thank you for your feedback.

2-8 Add wording that all matches at these tournaments were analyzed and if these represent males, females, or bot

**Response 3:**

We have added that it is men's tennis. It is true that it was not mentioned in the abstract. Thank you.

2-10 Clarify wording if these are means and what is “effectiveness?” Ball put in play or point won?

**Response 4:**

You are right. We did not specify that it is a point won. We have reflected that information in the Abstract. Thank you very much for your comment.

2-14 . . . won by the serving player? Medium rallies or longer?

**Response 5:**

We have modified this aspect in the abstract. We did not include information about longer length rallies to simplify the abstract. We can include it if you consider it appropriate. We wanted to highlight only the most relevant information from the study in this section.

2-17 Revise. You have no prospective evidence of tennis training. You have descriptive data of typical patterns of play in elite tennis players. This only informs what are typical and likely effective tactics and could inform coach and player judgments on training and match strategy

**Response 6:**

We have changed the wording of that sentence according to your comment.

**INTRODUCTION**

3-3 Revise this incorrect sentence. Like many sports, tennis has a long history of qualitative and quantitative performance analysis—most predating the automatic data collection systems seen recently in modern sport. This point is well presented in the Reid et al (5) article you cite, although they ignore classic texts in tennis (Classic textbooks by Talbert & Old, 1956, 1962, 1968, 1977 on singles and double tennis), early but influential tennis notation systems like COMPUTENNIS (Daw & Burton 1994; Kahn et al. 2004) and iCODA (O’Donoghue 2014; Wright et al. 2012). Your paper also cites quite a few more recent studies of notational data on tennis. The key observation here is that automated data collection and reporting systems has just accelerated recently because of these tools and growing sport/coaching awareness of the value of analytics. The first issue of the Journal of Sports Analytics was only 2015 and Int J Perform Analy Sport in 2001

**Response 7:**

Thank you for your very good comment. We have modified that sentence and incorporated the information you indicated.

3-11 to 13 Revise to clarify. Last sentence is good but next to last is an exaggeration/hyperbole. It is rare that any set of numbers can “optimize” training or performance. Even if such a complex system in a complex sport could be somewhat likely enhanced, in professional tennis there are limits on what a coach can communicate with players during a match

**Response 8:**

We have modified the penultimate sentence of the paragraph to avoid the hyperbole indicated. We agree that this is exaggerated information.

3-14 Revise this hyperbole: Delete implied and unobtainable optimality and focus on the search for meaningful variables for performance enhancement

**Response 9:**

Your comment is correct. We have changed the wording of the sentence. Perhaps optimise was not the most appropriate verb to use. Thank you for your suggestion.

3-22 Please stop with the meaningless hyperbole and one-sentence paragraph? What is optimal results in tennis? Winning every point in the match? Clearly communicate previous results and their meaning. How can a reader believe you that which stroke in tennis in any situation can be perfectly predicted, although it depends on many other things, and “in any case” the sport is just about the serve?!

**Response 10:**

We have modified the wording of the paragraph on the basis of your comment.

4-10 Vague wording. Different tournaments have different surfaces AND natural surfaces (clay & grass) have within tournament variation over time

**Response 11:**

We have improved the wording of this sentence in the document. Thank you for your input.

4-13 Revise for clarity. You are referring to the position on the court when a stroke is made . . . .shot direction and court position of the opponent

**Response 12:**

The wording of this sentence has been changed. Thank you for your comment.

4-18 Reword for clarity. Do you mean the chance of winning a point in a tennis match based on what or number of performance indicators?

**Response 13:**

We have reformulated the objective of the research on the basis of this comment. We believe it is more correct as you indicate.

**METHOD**

5-6 Analysis of only final rounds does not agree with wording in the abstract

**Response 14:**

We have modified the wording in the abstract to clarify this aspect also in that section.

5-11 Again, note if these are matches with males, females, or both. Also report the sample size since some players win and compete again, as well as may be in several tournament late rounds

**Response 15:**

We have added this information in the sample section.

6-Table 1 Correct tennis terminology (e.g., double fault). Perhaps have a table note with operational definitions of common tennis terms that may not be obvious to readers (e.g., forced versus unforced error)

**Response 16:**

We have corrected incorrect terminology. We have added a note at the end of the table with the definition of winner, forced error and unforced error. Thank you for your input.

7-4 Describe extent of training. Size and resolution of video, television used, and ability of observers to replay video sequences

**Response 17:**

This information now appears in the document. Thank you for your recommendation.

7-11 Vague. Please expand on what transformations were made and how this relates to the results of this previous study. Why are the results from reference 27 not summarized in the introduction (page 3)?

**Response 18:**

We have changed the wording of this paragraph. Reference 27 referred to a study where we had carried out a similar procedure but in another sport. As it is of little interest with the new wording of the paragraph, we removed the reference.

7-17 It is unclear how your rationale of a sequential analysis for this study aligns with your comparative analysis of tennis performance categorical data. Why is p < 0.05 is appropriate for this study given you have numerous Chi squared tests of likely correlated dependent variables. The family-wise type I error rate for this study is quite large, so you likely have some effects falsely identified as significant and you don’t know which ones are. An effective and easy approach is the progressive Holm (1979) correction

**Response 19:**

The sequential analysis we were referring to is an analysis of the server's probability of winning a point. We have redrafted that entire paragraph to explain in detail how that analysis was performed.

7-last line Describe this technique or cite a reference so readers can replicate your study

**Response 20:**

It is true that there was a lack of information to replicate the analysis. We have added information.

**RESULTS**

8-3 Good scientific reporting will have a narrative describing key results and parenthetically refer to the numerous specifics in a table. Combine this revised sentence with your results paragraphs

**Response 21:**

We have followed your commentary for the wording of the results in this revised version.

9-3 Is this correct? Inter-criterion for service was p = 0.085 and winners p = 0.373

**Response 22:**

We have carried out the analysis again and the results obtained were as shown in the table.

9-11 This paragraph mixes vague observations of nonsignificant and statistically significant

Response 23:

The results section has been modified as a whole. We have avoided non-significant comments.

10-9 Correct same error as line 8-3. Describe results and only parenthetically refer details in figure. Figure 2 needs a clear caption to facilitate reader understanding. This is also hard to understand given the vague presentation of what is meant by “effectiveness” and “sequential.” What is RG, WI, US?

**Response 24:**

Figure 2 had an error. Instead of RG (Roland Garros) it should read Clay, in Wi (Wimbledon) it should read Grass and in US (US Open) it should read hard. We have modified the figure. In addition, we have changed the wording of the paragraph before the figure to better explain how this figure should be interpreted.

11-10 through page 12 Consider condensing with only general results for interested readers to explore. The problem with you data are that they are likely biased by the handedness and players in these late rounds. It is hard to make sense of all these trends and what they might mean given the interaction with other factors (player, environment, match situation, handedness, etc)

**Response 25:**

In the revised version we have simplified the results. In addition, the information you indicate has been incorporated in a sub-section so that the reader can focus on the overall results. The other questions are added to the limitations section.

**DISCUSSION**

13-4 Readers should not be finding out in the discussion that the data only refer to male professional players

**Response 26:**

Thank you for your comment. It is true that there was little indication that the study was on men. In the revised paper the reference to men's tennis appears in the title (also in the original version), in the abstract (the original version did not include), in the method (the original version did not include) and in results (the original version did not include).

13-6 Do not repeat your reporting of means by surface. The report improved by integrating this idea with your reporting of previous values

**Response 27:**

The discussion has been drafted in this revision following your advice. Thank you for your comment.

13-19 . . . first serves might be an effective match strategy. The current data indicate that this might increase points early between 14 and 21% for elite players depending on the surface

**Response 28:**

We have changed the meaning of the sentence. It now appears as you suggest.

13- last line Do not refer just the last few years. The speed of tennis has been increasing for many years due to improvements in racket design (Haake & Brody 1999; Haake et al. 2000, 2007) and was even considered slowing by changes (type III ball)

**Response 29:**

We have taken this comment into account and changed the text in the document. Thank you for your input.

14-5 Unclear. What does it mean to look for a winner?

**Response 30:**

We have changed the meaning of the phrase to make it more understandable.

We have asked American tennis coaches if this expression is correct and they indicated that it is. In any case, we welcome suggestions to modify the expression in case it is not clear.

14-8 What does it mean to look for an unforced error?

**Response 31:**

Same as in the previous comment.

**CONCLUSION**

16-5 Add additional limitation of small number of players and potential interaction on fatigue/stress of additional rounds of winners that are apparently treated as independent matches/observations and are not. Weakness of inflation of type I errors also needs to be noted if not controlled

**Response 32:**

We have added this information in the limitations. They now appear after the conclusions at the suggestion of the other reviewer. Thank you for your comment.

Conclusions: Nice summary. Perhaps reword to chance of winning a point rather than “effectiveness” and other wording for a “final shot” (aggressive/challenging second shot)

**Response 33:**

Thank you for your comment. We have modified the text according to your feedback.
