# Supplementary material for: Match analysis and probability of winning a point in elite men’s singles tennis
Source: PLoS One. 2023 Sep 28;18(9):e0286076. doi: 10.1371/journal.pone.0286076 (PMC10538650; doi:10.1371/journal.pone.0286076)
Supplement: Attachment — Submitted filename: 2023 - Match analysis and probability - Crespo 2nd review.docx [file pone.0286076.s006.docx]

**Match analysis and probability of winning a point in men's tennis.**

**2^nd^ review**

**Reviewer:** Miguel Crespo, International Tennis Federation

**Introduction**

The reviewer would like to thank the authors for their efforts on this second version of the manuscript. In the reviewer's opinion, so many changes have been made that it really does look like a new document.

In addition, the reviewer acknowledges that the authors have considered many of the suggestions made in the first revision.

Similarly, the reviewer has noted that the authors have also taken into account many of the suggestions made by the second reviewer.

It is also noted that the authors have made considerable improvements in the English language, which makes the manuscript easier to understand.

However, despite the improvements made and the fact that the changes are so substantial that a new text could be considered, this reviewer considers that, without detracting from the interest of the work, there are still aspects that could be clarified and improved.

Therefore, in this second revision, the reviewer proposes to continue the academic debate raised by this new version in order to improve the manuscript as much as possible.

To this end, the following paragraphs are intended to assist in the improvement and development of the text presented.

**Abstract**

The authors have changed the abstract considerably but, in the reviewer's opinion, there are still some aspects that are unclear and contribute to a general confusion that does little to aid understanding of the text.

For example, they start by talking about tactical actions in tennis and then include the term technical-tactical performance factors. They then discuss the most important performance indicators in the sport. Obviously, the mention of three very similar terms, all related to each other, does not help to clearly understand what the authors intend. It seems as if they want to follow the well-known phrase: "If you can't convince them, confuse them".

And that is what happens to this reviewer with this new version in general. A constant confusion that, added to the lack of objectives and hypotheses, produces a sense of unfamiliarity and general lack of precision that does not help the manuscript.

In some sentences, such as "The end of the point (winner, forced error and unforced error) varies according to the court surface, the type of serve and the length of the rally" the obviousness is clear and probably does not need to be included.

Moreover, the final sentence is so ambiguous and general that it needs to be more specific to give practical value to the results of the study.

**Introduction - KPIs**

The changes made in the first part of the introduction, some of them suggested by the other reviewer, are appropriate and improve the quality of the previous version.

However, having reread this introduction and, in particular, the second part where the authors should go into detail about the research carried out on the subject of their study, this reviewer notes a lack of depth in the reference to the results of this research.

And this is where the reviewer notes a general confusion regarding the concept of key performance indicator. In this case, it seems that the authors consider serve and court surface to be key performance indicators in the same way as rally duration.

With all due respect, this reviewer believes that this is where the major confusion in this study lies. The serve, like the rest, the forehand or any other stroke, are technical actions or gestures. They can in no way be considered key performance indicators.

Similarly, the court surface, as well as the height of the court (at sea level or at altitude), or whether it is an indoor or outdoor court, or even the type of balls with which the match is played, cannot be considered as key performance indicators.

The fact that the authors have eliminated the different terms used to refer to what they call study variables (patterns, combinations of blows, behaviours, etc.) does not mean that the terminological confusion is not present throughout the text and should therefore be clarified and suitably modified to prevent the study, its approach, results and conclusions from being unclear and imprecise.

Similarly, when they refer to combinations of strokes (the so-called patterns), as in the case of the serve + a stroke, for example, they are not key performance indicators either, but rather patterns of play (obviously tactical) made up of combinations of gestures, but not KPIs.

**Objective and hypotheses**

With regard to the lack of hypotheses in the study, the authors justify this fact by the type of study in question. However, once again, in the opinion of this reviewer, if the authors do not wish to include hypotheses, which is regrettable considering the large number of previously conducted studies to which they could refer and from which to obtain possible hypotheses to validate in their research, they should at least include more objectives that would help them to structure the results and discussion section much better.

In this sense, the reviewer considers, once again, that, lacking clear, specific hypotheses based on the results of previous studies, which are not taken as a basis for this observational study, the research we reviewed lacks reference points as only one objective is established.

Again, with respect, the reviewer feels that in light of the results of previous research, the authors should set many more objectives to inform their study. In fact, a mention to the results of these previous studies should be included in the last section of the introduction to provide the necessary background to the study and to show that the authors have done the adequate homework in their literature study by extracting the main results of these previous works.

As they indicate in the new version “Based on the above, the objectives of the research were to analyse the technical-tactical performance indicators of men's Grand Slam tennis matches, as well as to study the probability of winning a point”, if these are the two objectives of the study, they should be stated more specifically based on the results of research previously obtained.

**Methodology – instruments**

It is important to note that the instrument used is, as stated by the authors, a system of categories. Again, this is a new term. Does this mean that categories are key performance indicators? Are they game situations? Are they patterns? Are they shots? The confusion continues and it does not seem to be clarified.

Regarding the validation of the instrument, the reviewer is unsure if the feedback of two tennis experts is enough to validate such a tool.

Furthermore, in the table 1 the confusion continues because the categories are then called “criteria”… thus not helping to clarify the overall confusion of terminologies.

**Results**

Again, when the authors state that they are analyzing technical-tactical performance indicators, there are several major issues: as indicated, the service is not a performance indicator, in the same way that the zone of the court in which the ball bounces, or where the ball is played to…

The titles, such as the probability of winning a point based on different combinations of performance indicators is again misleading, because the serve is not a performance indicator, nor the court surface, or zone of ball bounce…

**Discussion**

As indicated above, the results of these previous studies should be used as the main base to generate not only the structure of the results but also that of the discussion. This would help to provide the necessary clarity to the study and to show that the authors have done the adequate homework in their literature study by extracting the main results of these previous works and, once they have their own results, discussing adequately with those of their colleagues.

**Conclusion**

Again, the authors refer to the service, surface, and rally length. Two of these aspects are not key performance indicators, and this should be clarified.

The practical application section is extremely short, quite poor in content and suggestions for practitioners and with an obvious lack of belief in the applicability of the results obtained. It would be convenient to cover this with more depth and with a clear intention of sharing the positive aspects of the study.

**Final words**

Again, as in the first review, the reviewer would like to thank the authors for their interest in conducting studies on tennis. The reviewer hopes that the comments made in this review on the second version of the manuscript will be useful to the authors.

The fact that so many changes have been made has, on the one hand, helped to improve the English. However, on the other hand, new content, expressions, and terms have been introduced which may have contributed to confusion and opacity of the text.

The reviewer hopes that the authors receive these comments on the second version of the text as an attempt to improve the quality of the text and as part of the process involved in any scientific publication.

Once again, the reviewer's intention is to help the manuscript improve its quality and reach the appropriate level to be published in the journal. At the moment, this reviewer's recommendation is that the authors make considerable improvements to it.

Best regards,

Miguel Crespo, PhD.

International tennis Federation
