# Supplementary material for: Match analysis and probability of winning a point in elite men’s singles tennis
Source: PLoS One. 2023 Sep 28;18(9):e0286076. doi: 10.1371/journal.pone.0286076 (PMC10538650; doi:10.1371/journal.pone.0286076)
Supplement: Attachment — Submitted filename: Response to reviewers.docx [file pone.0286076.s007.docx]

**Reviewer #1: Match analysis and probability of winning a point in men's tennis.**

**2nd review**

**Reviewer: Miguel Crespo, International Tennis Federation**

The reviewer would like to thank the authors for their efforts on this second version of the manuscript. In the reviewer's opinion, so many changes have been made that it really does look like a new document.
In addition, the reviewer acknowledges that the authors have considered many of the suggestions made in the first revision.
Similarly, the reviewer has noted that the authors have also taken into account many of the suggestions made by the second reviewer.
It is also noted that the authors have made considerable improvements in the English language, which makes the manuscript easier to understand.
However, despite the improvements made and the fact that the changes are so substantial that a new text could be considered, this reviewer considers that, without detracting from the interest of the work, there are still aspects that could be clarified and improved.
Therefore, in this second revision, the reviewer proposes to continue the academic debate raised by this new version in order to improve the manuscript as much as possible.
To this end, the following paragraphs are intended to assist in the improvement and development of the text presented.

Response

Once again, thank you very much for your review. It has certainly helped to improve the article. We have made an effort to introduce all the changes requested in this review.

**Abstract**

The authors have changed the abstract considerably but, in the reviewer's opinion, there are still some aspects that are unclear and contribute to a general confusion that does little to aid understanding of the text.
For example, they start by talking about tactical actions in tennis and then include the term technical-tactical performance factors. They then discuss the most important performance indicators in the sport. Obviously, the mention of three very similar terms, all related to each other, does not help to clearly understand what the authors intend. It seems as if they want to follow the well-known phrase: "If you can't convince them, confuse them".
And that is what happens to this reviewer with this new version in general. A constant confusion that, added to the lack of objectives and hypotheses, produces a sense of unfamiliarity and general lack of precision that does not help the manuscript.
In some sentences, such as "The end of the point (winner, forced error and unforced error) varies according to the court surface, the type of serve and the length of the rally" the obviousness is clear and probably does not need to be included.
Moreover, the final sentence is so ambiguous and general that it needs to be more specific to give practical value to the results of the study.

**Response**

We have taken into account the comments in this section. To avoid confusion of terms, we have modified the terminology. In this review we will refer to "variables influencing performance". It is true that not all the variables studied are technical-tactical indicators, such as the court surface. But, without a doubt, the surface is a variable that influences the way the point is played.

We have changed the ambiguous sentence you indicated for a more specific one about the results of the research. On the other hand, we have stated a number of hypotheses to guide the study as you suggest.

**Introduction - KPIs**

The changes made in the first part of the introduction, some of them suggested by the other reviewer, are appropriate and improve the quality of the previous version.
However, having reread this introduction and, in particular, the second part where the authors should go into detail about the research carried out on the subject of their study, this reviewer notes a lack of depth in the reference to the results of this research.
And this is where the reviewer notes a general confusion regarding the concept of key performance indicator. In this case, it seems that the authors consider serve and court surface to be key performance indicators in the same way as rally duration.
With all due respect, this reviewer believes that this is where the major confusion in this study lies. The serve, like the rest, the forehand or any other stroke, are technical actions or gestures. They can in no way be considered key performance indicators.
Similarly, the court surface, as well as the height of the court (at sea level or at altitude), or whether it is an indoor or outdoor court, or even the type of balls with which the match is played, cannot be considered as key performance indicators.
The fact that the authors have eliminated the different terms used to refer to what they call study variables (patterns, combinations of blows, behaviours, etc.) does not mean that the terminological confusion is not present throughout the text and should therefore be clarified and suitably modified to prevent the study, its approach, results and conclusions from being unclear and imprecise.
Similarly, when they refer to combinations of strokes (the so-called patterns), as in the case of the serve + a stroke, for example, they are not key performance indicators either, but rather patterns of play (obviously tactical) made up of combinations of gestures, but not KPIs.

**Response**

As indicated above, in order to avoid that the terminology used may confuse the reader, we have modified the entire text. In this version we will refer to variables that influence the outcome of the point by eliminating the performance indicator terminology. We will use the term pattern of play in some cases, but for the most part we have chosen to talk about a combination of variables that influence the outcome of the point.

**Objective and hypotheses**

With regard to the lack of hypotheses in the study, the authors justify this fact by the type of study in question. However, once again, in the opinion of this reviewer, if the authors do not wish to include hypotheses, which is regrettable considering the large number of previously conducted studies to which they could refer and from which to obtain possible hypotheses to validate in their research, they should at least include more objectives that would help them to structure the results and discussion section much better.
In this sense, the reviewer considers, once again, that, lacking clear, specific hypotheses based on the results of previous studies, which are not taken as a basis for this observational study, the research we reviewed lacks reference points as only one objective is established.
Again, with respect, the reviewer feels that in light of the results of previous research, the authors should set many more objectives to inform their study. In fact, a mention to the results of these previous studies should be included in the last section of the introduction to provide the necessary background to the study and to show that the authors have done the adequate homework in their literature study by extracting the main results of these previous works.
As they indicate in the new version “Based on the above, the objectives of the research were to analyse the technical-tactical performance indicators of men's Grand Slam tennis matches, as well as to study the probability of winning a point”, if these are the two objectives of the study, they should be stated more specifically based on the results of research previously obtained.

**Response**

A total of five hypotheses have been proposed, all of them with a previous theoretical basis. Both the results and the discussion are written on the basis of the objectives and hypotheses proposed.

**Methodology – instruments**

It is important to note that the instrument used is, as stated by the authors, a system of categories. Again, this is a new term. Does this mean that categories are key performance indicators? Are they game situations? Are they patterns? Are they shots? The confusion continues and it does not seem to be clarified.
Regarding the validation of the instrument, the reviewer is unsure if the feedback of two tennis experts is enough to validate such a tool.
Furthermore, in the table 1 the confusion continues because the categories are then called “criteria”… thus not helping to clarify the overall confusion of terminologies.

Response

The observational instrument was always a system of categories, a term commonly used by observational methodology. It is true that the "criteria" are not indicators of performance, but variables or factors affecting performance. We have modified that in the table.

In observational methodology it is common for variables to be called criteria. In any case, the term has been changed and we will use variables.

It is true that the more experts validating the instrument the better, but observational methodology itself considers it appropriate to use at least two. Moreover, in our case it was two experts in observational methodology and tennis at the same time.

**Results**

Again, when the authors state that they are analyzing technical-tactical performance indicators, there are several major issues: as indicated, the service is not a performance indicator, in the same way that the zone of the court in which the ball bounces, or where the ball is played to…
The titles, such as the probability of winning a point based on different combinations of performance indicators is again misleading, because the serve is not a performance indicator, nor the court surface, or zone of ball bounce…

Response

You are right. For this reason, as indicated above, we have made a change in terminology to avoid confusion. We believe that if we talk about variables that affect performance or variables that influence the outcome of the point we will avoid this terminological confusion by mixing variables from different fields.

**Discussion**

As indicated above, the results of these previous studies should be used as the main base to generate not only the structure of the results but also that of the discussion. This would help to provide the necessary clarity to the study and to show that the authors have done the adequate homework in their literature study by extracting the main results of these previous works and, once they have their own results, discussing adequately with those of their colleagues.

Response

The results and discussion section has been modified to take into account the hypotheses. We hope that this has now been done as requested.

**Conclusion**

Again, the authors refer to the service, surface, and rally length. Two of these aspects are not key performance indicators, and this should be clarified.
The practical application section is extremely short, quite poor in content and suggestions for practitioners and with an obvious lack of belief in the applicability of the results obtained. It would be convenient to cover this with more depth and with a clear intention of sharing the positive aspects of the study.

Response

We have varied the language throughout the document to remove the performance indicator term from the previous version. Regarding the practical implications section, we have tried to follow the suggestions of both reviewers, but the recommendations are not along the same lines.

**Final words**

Again, as in the first review, the reviewer would like to thank the authors for their interest in conducting studies on tennis. The reviewer hopes that the comments made in this review on the second version of the manuscript will be useful to the authors.
The fact that so many changes have been made has, on the one hand, helped to improve the English. However, on the other hand, new content, expressions, and terms have been introduced which may have contributed to confusion and opacity of the text.
The reviewer hopes that the authors receive these comments on the second version of the text as an attempt to improve the quality of the text and as part of the process involved in any scientific publication.
Once again, the reviewer's intention is to help the manuscript improve its quality and reach the appropriate level to be published in the journal. At the moment, this reviewer's recommendation is that the authors make considerable improvements to it.

Best regards,

Miguel Crespo, PhD.
International tennis Federation

Response

Thank you very much for all the comments and recommendations. The manuscript has undoubtedly improved thanks to your input.

Iván Prieto Lage

Universidade de Vigo

**Reviewer #2:**

The authors did a generally good job addressing the points of the initial review and the data on elite male players in singles tennis are valuable. They did not cite all the recommended articles or the older COMPUTENNIS data/Talbert & Old texts on patterns of tennis play. There is a need for clarity in focus on elite males singles (I presume singles rather than both singles and doubles) and reporting of “percentage points” or actual percentage differences. The revision still does not clearly communicate that the patterns and error rates are specific only to elite, male tennis players in singles match play. Please consider the revisions noted in the specific comments that follow.

**Page-Line**

**Comment 1**

2-1 . . . in elite men’s singles tennis

Response:

Thank you for your suggestion. We have changed the title according to your recommendation.

**Comment 2**

2-9

Response:

This comment was blank. It must have been a mistake.

**Comment 3**

2-20 . . . on match strategy at the highest levels of elite men’s single tennis.

Response:

We have added the information you requested. Thank you for your suggestion.

**Comment 4**

4-63 There should not be any one-sentence paragraphs. Consider revising to have a sentence with both objectives of tactical performance indicators and probability of winning at point in elite-level men’s tennis followed by a statement of importance. Something like: Data from the late rounds of three of four Grand Slam men’s singles tournaments allow the documentation of the tactical demands and success rates at the highest level of play of the sport.

Response:

Thank you for your comment. We have taken on board your suggestion which will be combined with the other reviewer's suggestion regarding the formulation of hypotheses.

**Comment 5**

14-245 to 311 Please revise carefully to clarify when you are talking about differences in error rates/percentages. For example, the wording should be: . . . “the probability of winning a point decreases 25 to 29 percentage points” if you are just subtracting error rates. When you calculate a percentage it is essential to know what the denominator is for the percentage calculation. So if you do not define this and just are subtracting percentages in different conditions, you have to clearly state that there is X percentage point difference, NOT at some percentage difference using some unspecified comparison condition

Response:

We have amended the text in the light of this comment. Thank you for your recommendation.

**Comment 6**

14-245 to 248 Delete redundant paragraph repeating the purpose of your study. Lead your discussion off with the most important observation(s) and their size/strategic meaning. Again, the author(s) should note that the results represent the highest level of performance in the sport—near and earning championship at the men’s elite leve1

Response:

We have reworked the introduction of the discussion and specified that this is a study on elite men's singles tennis.

**Comment 7**

14-249 to 253 Good comparison, but can there be more context added? Are previous studies of advanced or elite levels and both sexes?

Response:

You are right. The studies we compare it to are from the same context (elite men's singles tennis). We have added this information to the text.

**Comment 8**

17-315 . . . to the first serve in elite men’s singles tennis. Other option, perhaps better option, is to set up these conclusions from your data that relate to elite men’s singles tennis play.

Response:

We have taken up the suggestion and it has been added to the document.

**Comment 9**

17-318 . . . most common point winning combination of shot for the server was.

Response:

Thank you for your comment. We have modified the wording of the text.

**Comment 10**

17-324 Revise to eliminate “guidelines.” You have general benchmarks for elite-level men’s tennis. You have no idea how different skill levels, disabilities, or sex of player might influence the pattens of play and error rates

Response:

We have changed the wording of the paragraph.

**Comment 11**

17-329 How should coaches use this? Just say that these patterns and success/errors rates are what are currently present at the highest levels. Male players seeking this level of play should consider training and shots in match play that align with greater probability of success

Response:

Thank you for your feedback. We have included your suggestion in the document.

**Comment 12**

18-344 Add a sentence that the stroke patterns and error rates observed may not be similar for players from other sex, skill level, or disabilities.

Response:

That is a good point. Thank you. It has been added to the text.
